# Repurposing screen identifies novel candidates for broad-spectrum coronavirus antivirals and druggable host targets

**DOI:** 10.1128/aac.01210-23

**Published:** 2024-02-06

**Authors:** Sibylle Haid, Alina Matthaei, Melina Winkler, Svenja M. Sake, Antonia P. Gunesch, Vanessa Milke, Natalie M. Köhler, Jessica Rückert, Gabrielle Vieyres, David Kühl, Tu-Trinh Nguyen, Matthias Göhl, Lisa Lasswitz, Francisco J. Zapatero-Belinchón, Graham Brogden, Gisa Gerold, Bettina Wiegmann, Ursula Bilitewski, Richard J. P. Brown, Mark Brönstrup, Thomas F. Schulz, Thomas Pietschmann

**Affiliations:** 1 Institute for Experimental Virology, Twincore - Centre for Experimental and Clinical Infection Research, Hannover, Germany; 2 Institute of Virology, Hannover Medical School, Hannover, Germany; 3 German Center for Infection Research, Hannover-Braunschweig Site, Hannover, Germany; 4 Junior Research Group “Cell Biology of RNA Viruses”, Leibniz Institute of Experimental Virology, Hamburg, Germany; 5 Integrative Analysis of Pathogen-Induced Compartments, Leibniz ScienceCampus InterACt, Hamburg, Germany; 6 Calibr, a Division of The Scripps Research Institute, La Jolla, California, USA; 7 Helmholtz Centre for Infection Research, Braunschweig, Germany; 8 Department of Biochemistry & Research Center for Emerging Infections and Zoonoses (RIZ), University of Veterinary Medicine Hannover, Hannover, Germany; 9 Department of Clinical Microbiology, Virology, 901 87 Umeå University, Umeå, Sweden; 10 Wallenberg Centre for Molecular Medicine (WCMM), 901 87 Umeå University, Umeå, Sweden; 11 Cluster of Excellence RESIST (EXC 2155), Hannover Medical School, Hannover, Germany; 12 Department of Cardiothoracic, Transplantation and Vascular Surgery, Hannover Medical School, Hannover, Germany; 13 Lower Saxony Center for Biomedical Engineering, Implant Research and Development, Hannover Medical School, Hannover, Germany; 14 BREATH (Biomedical Research in Endstage and Obstructive Lung Disease Hannover), German Center for Lung Research (DZL), Carl-Neuberg Str. 1, Hannover, Germany; 15 Division of Veterinary Medicine, Paul Ehrlich Institute, Langen, Germany; 16 Department of Molecular and Medical Virology, Ruhr University, Bochum, Germany; IrsiCaixa Institut de Recerca de la Sida, Badalona, Spain

**Keywords:** antivirals, coronavirus, HCoV-229E, repurposing, SARS-CoV-2, host-targeting antiviral therapy, CRISPR/Cas9

## Abstract

Libraries composed of licensed drugs represent a vast repertoire of molecules modulating physiological processes in humans, providing unique opportunities for the discovery of host-targeting antivirals. We screened the Repurposing, Focused Rescue, and Accelerated Medchem (ReFRAME) repurposing library with approximately 12,000 molecules for broad-spectrum coronavirus antivirals and discovered 134 compounds inhibiting an alphacoronavirus and mapping to 58 molecular target categories. Dominant targets included the 5-hydroxytryptamine receptor, the dopamine receptor, and cyclin-dependent kinases. Gene knock-out of the drugs’ host targets including cathepsin B and L (CTSB/L; VBY-825), the aryl hydrocarbon receptor (AHR; Phortress), the farnesyl-diphosphate farnesyltransferase 1 (FDFT1; P-3622), and the kelch-like ECH-associated protein 1 (KEAP1; Omaveloxolone), significantly modulated HCoV-229E infection, providing evidence that these compounds inhibited the virus through acting on their respective host targets. Counter-screening of all 134 primary compound candidates with SARS-CoV-2 and validation in primary cells identified Phortress, an AHR activating ligand, P-3622-targeting FDFT1, and Omaveloxolone, which activates the NFE2-like bZIP transcription factor 2 (NFE2L2) by liberating it from its endogenous inhibitor KEAP1, as antiviral candidates for both an *Alpha*- and a *Betacoronavirus*. This study provides an overview of HCoV-229E repurposing candidates and reveals novel potentially druggable viral host dependency factors hijacked by diverse coronaviruses.

## INTRODUCTION

The last two decades have been marked by epidemics and pandemics caused by emerging viruses within the family *Coronaviridae*, leaving devastating consequences for both our global health and economics. With unclear pathogenic and pandemic potential of existing coronaviruses and a lack of understanding of determinants of cross-species transmission, we inevitably face future outbreaks initiated by new and genetically diverse coronaviruses.

**Fig 1:**
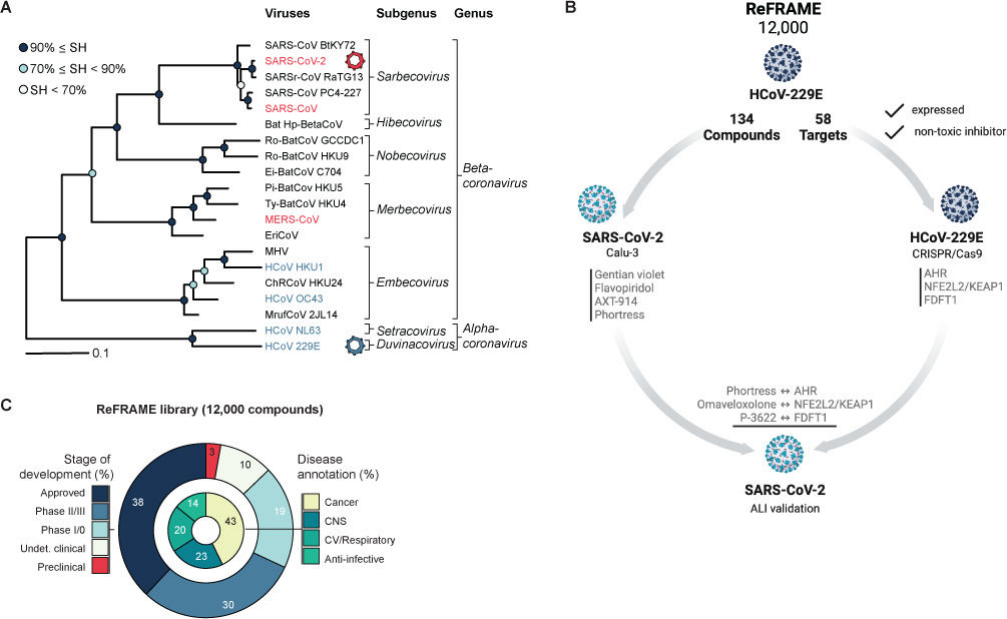
Genetic diversity of human pathogenic coronaviruses, ReFRAME library composition, and screening workflow. (**A**) A section of the coronavirus phylogeny based on an IQ-TREE maximum-likelihood tree showing representative viruses of 20 species representing all 18 species of the genus *Betacoronavirus* and two of the genus *Alphacoronavirus* (adapted from (1)). Branch support was estimated using the Shimodaira-Hasegawa (SH)-like approximate likelihood ratio test with 1,000 replicates (SH = sequence homology). (**B**) Workflow of our approach to simultaneously identify broad-spectrum coronavirus antivirals and host targets thereof. Created with BioRender.com. (**C**) Composition of the ReFRAME drug repurposing library.

The subfamily *Orthocoronavirinae* is composed of four genera: *Alpha*-, *Beta*-, *Gamma*-, and *Deltacoronavirus* (Fig. 1A). The viruses of special concern, severe acute respiratory syndrome coronavirus 1 (SARS-Co-V) and SARS-CoV-2 belong to the subgenus *Sarbecovirus* of the genus *Betacoronavirus*, whereas Middle East respiratory syndrome coronavirus (MERS-CoV) belongs to the subgenus *Merbecovirus*, also within the *Betacoronavirus* genus. Other seasonal coronaviruses which circulate in human populations and display milder respiratory and/or intestinal disease include the *Betacoronaviruses* human coronaviruses (HCoV) HCoV-HKU1 and HCoV-OC43 (subgenus: *Embecovirus*), and the *Alphacoronaviruses* HCoV-NL63 (subgenus: *Setracovirus*) and HCoV-229E (subgenus: *Duvinacovirus*) (Fig. 1A) (1). With the widespread taxonomic distribution of these human respiratory pathogens and the existence of other coronaviruses with unknown zoonotic potential, it is vital to develop broad-acting antiviral drugs that target divergent coronavirus species. Therefore, special focus must be put upon the development of novel and innovative antiviral strategies as a crucial strategy for pandemic preparedness.

Eventhough numerous highly effective vaccine candidates have been developed for the reduction of SARS-CoV-2 infections and the risk of severe disease, protection from seasonal coronaviruses varies (2). Furthermore, natural immunity and vaccine-induced immunity typically remain transient with a remaining risk of breakthrough infections (3, 4). Specific antiviral treatment options against coronaviruses remain sparse (5): The authorization of monoclonal antibody treatments, such as bamlanivimab (LY-CoV555) and etesevimab (LY-CoV016), used for the treatment of mild to moderate SARS-CoV-2 infections in the early stages of the COVID-19 pandemic has since been revoked by the FDA, reporting the emergence of resistance mutations in newly emerging SARS-CoV-2 variants (6–8). Remdesivir, a broad-spectrum nucleoside analog, inhibits the replication of SARS-CoV-2 and was licensed for the treatment of severe COVID-19. However, its efficacy for the treatment of SARS-CoV-2 infections appears to be moderate based on differential outcomes of clinical trials and the World Health Organization (WHO) no longer recommends its use in COVID-19 patients (9).

Recently, orally administered antiviral compounds Lagevrio (molnupiravir) and Paxlovid (nirmatrelvir and ritonavir) have been granted emergency or regular authorization by the FDA for the treatment of mild-to-moderate COVID-19 disease (10, 11). Paxlovid combines the main protease inhibitor nirmatrelvir, with ritonavir, a CYP3A inhibitor, and human immunodeficiency virus 1 (HIV-1) protease inhibitor, and has shown to be effective in reducing the mortality and hospitalization rates in patients with COVID-19. Although early studies have shown the effectiveness of nirmatrelvir against other seasonal coronaviruses additional in-depth research is required (12). Molnupiravir is a nucleoside analog that inhibits SARS-CoV-2 replication by viral mutagenesis (13). Here, studies have shown a significant reduction in the risk of hospital admission or death (14). However, no significant benefit has been observed when administering Molnupiravir in a later stage of moderate-to-severe COVID-19 (15). Studies on the efficacy of this antiviral against additional coronaviruses are still lacking at this point (14).

As intracellular parasites, viruses hijack host cellular co-factors to replicate. Targeting this dependency, host-directed treatments (HDTs) aim to halt the viral replication cycle by blocking these crucial host factors.

Successful and commercially available examples include host-directed CC chemokine (CCR5) receptor inhibitors, such as maraviroc, which block HIV-1 entry (16, 17), or the recently licensed NTCP-inhibitor Myrcludex B (Hepcludex), which inhibits the entry of hepatitis D and hepatitis B virus into host cells (18). HDTs targeting these host factors may be useful for the development of mutation-resistant antiviral therapies, as viruses are less prone to mutate during the duration of treatment and are prone to adapt only under long-term selection pressures (19). Furthermore, as viral families frequently exploit similar cellular pathways, the discovery of broad-spectrum HDTs would facilitate the treatment of future and previously emerging and re-emerging viruses (19).

Recently, studies have identified shared host factor networks across all coronaviruses (20). However, there is an unmet need for comprehensive screens focusing on druggable pathways as well as on candidates for broad-spectrum anti-coronavirus therapeutics.

The Repurposing, Focused Rescue, and Accelerated Medchem (ReFRAME) is a drug repurposing library composed of about 12,000 small molecules (Fig. 1B) (21). Around 68% of these compounds are either licensed for use in humans or advanced stages of clinical development (Fig. 1B). Disease annotations for physiological processes targeted by these molecules are dominated by cancer (43%), central nervous system (CNS, 23%), and cardiovascular/respiratory (20%). Therefore, 86% of molecules in the ReFRAME library target physiological processes in humans (https://reframedb.org). The composition and breadth of this library showcase a unique resource for the identification of molecules targeting specific and well-defined host pathways and factors with potential for the development of broad-spectrum HDTs. Recent screening efforts using the ReFRAME library against SARS-CoV-2 revealed promising drug repurposing candidates (3, 22–24).

In our current study, we utilize the ReFRAME library to (1) identify drugs and drug-like molecules with therapeutic potential against two genetically diverse coronaviruses and (2) conduct a CRISPR/Cas9 knock-out screen for the consolidation of selected hit compounds and their host targets, highlighting an innovative way for the discovery of molecules targeting host factors and pathways for the potential use as broad-spectrum HDTs of coronaviruses (Fig. 1C).

## MATERIALS AND METHODS

### Cell lines

The following cell lines were used for this study: Vero cells (*Chlorocebus aethiops*, ATCC-CCL-81; Lot 58484194), Vero E6 cells (*Chlorocebus aethiops*) and Calu-3 cells (*Homo sapiens sapiens*) (both from Stefan Pöhlmann), Huh-7.5 cells (*Homo sapiens sapiens*, Charles M. Rice), MRC-5 cells (*Homo sapiens sapiens*, Volker Thiel), HEK 293T cells (*Homo sapiens sapiens*, ATCC CCL-3216), 16HBE140 cells (*Homo sapiens sapiens*, Gert Zimmer), A549 cells (*Homo sapiens sapiens*, ATCC-CCL-185; Lot 59239596), HEp-2 (*Homo sapiens sapiens*, ATCC-CCL-23; Lot 58978772), and A427 cells (*Homo sapiens sapiens*, cell line services CLS# 300111; Lot 300111–612). Huh-7.5/F-Luc cells were created by lentiviral gene transfer using a pWPI-vector encoding a firefly luciferase (F-Luc) transgene under the control of an elongation factor 1-alpha promotor and a blasticidine drug resistance gene. Finally, Huh-7.5/F-Luc/Cas9 cells were created by gene transfer from the Cas9-encoding vector pLKO5d.EFS.SpCas9.P2A.BSD (25) into Huh-7.5/F-Luc cells. pLKO5d.EFS.SpCas9.P2A.BSD was a gift from Benjamin Ebert (Addgene plasmid # 57821; http://n2t.net/addgene:57821; RRID: Addgene_57821).

### Virus propagation

Vero cells (ATCC-CCL-81; Lot 58484194) were cultured in T75 flasks in Advanced Minimal Essential Medium supplemented with 1% (vol/vol) non-essential amino acids (NEAA), 100 U/mL penicillin, 100 µg/mL streptomycin, 2 mM L-glutamine and 10% FCS at 37°C (5% CO_2_). At 50%–80% confluency, Vero cells were inoculated with 500 µL of SARS-CoV-2 (strain SARS-CoV-2/München-1.2/2020/984; p3) in a total of 10 mL of Advanced MEM and incubated at 37°C (5% CO_2_) under BSL-3 conditions adapted for infectious respiratory viruses. As soon as cytopathic effects were visible, mostly at 96 hours post-infection (hpi), the supernatant was collected, centrifuged at 1,000 × *g* for 10 min, and stored in aliquots at −80°C. Stocks were titrated on Vero and Calu-3 (26) cells (ATCC Cat. #HTB-55 and RRID: CVCL_0609, respectively), cultured in DMEM containing 1% NEAA, 100 U/mL penicillin, 100 µg/mL streptomycin, 2 mM L-glutamine, and 10% FCS. At 72 hpi, cells were fixed with 10% formalin for 30 min and stained with crystal violet. Viral titer (TCID_50_/mL) was quantified based on the Spearman and Karber method using a calculator developed by Marco Binder, Heidelberg University. HCoV-229E/R-Luc stocks (27) (kind gift of Volker Thiel, University of Bern, Switzerland) were prepared by inoculation of Huh-7.5 cells. After 48 hours of incubation at 33°C, virus-containing supernatant was harvested and cleared from cell debris by centrifugation at 1,000 × *g* for 10 min prior to storage at −80°C. Titration of the HCoV-229E/R-Luc stock was done by limiting dilution assay (TCID_50_/mL) using a primary antibody against HCoV-229E N-protein (Ingenasa #M.30.HCo.I1E7; Lot250609; final concentration 2 µg/mL) based on the method of Spearman and Karber.

### HCoV-229E screening

Huh-7.5/F-Luc cells were seeded in black 384-well plates with a flat clear bottom (Corning #3764) at a density of 3 × 10^3^ cells/well in phenol-red free Dulbecco’s modified Eagle medium (DMEM; Gibco #31053–028) supplemented with 2 mM L-glutamine (Gibco #25030024), 100 µg/mL streptomycin and 100 U/mL penicillin (Gibco #15140122), 10% fetal calf serum (FCS, Capricorn Scientific #FBS-11A), and 1% (vol/vol) non-essential amino acids (NEAA; Gibco #11140035) 1 day prior to infection. Cells were infected with an HCoV-229E *Renilla* luciferase reporter virus at an MOI of 0.1 in the presence of a final drug concentration of 5 µM using a Beckman Coulter I5 automated workstation. Blinding was done by Calibr by providing ID numbers instead of compound names. The Scripps Research Institute quality controlled the compounds contained in the ReFRAME library *via* liquid chromatography-mass spectrometry (LC-MS) and nuclear magnetic resonance spectroscopy ^1^H-(NMR) to acquire a purity of ≥95% (21). Following incubation for 48 hours at 33°C, luciferase activity was determined using the dual-glo luciferase assay system (Promega, #E2920) and a GloMax machine (Promega; GM3000; software version 3.1.0) according to the instructions of the manufacturer. In brief, 60 µL of cell culture fluid was removed from each well to obtain a final volume of 20 µL per well prior to the addition of 20 µL of Dual-glo luciferase assay reagent, incubation for 20 minutes at room temperature and subsequent firefly luciferase measurement (0.5-s detection time). Firefly luminescence was quenched by the addition of 20 µL per well Dual-glo stop & glo reagent. After an additional 20-minute incubation at room temperature, *Renilla* luminescence was analyzed (0.5-s detection time).

### CRISPR/Cas9 screen with HCoV-229E in Huh-7.5/F-Luc/Cas9 cells

For the knock-out of host cellular target factors, plasmids encoding the specific single guide RNAs (sgRNAs) and a dTomato reporter gene using the SGL40C.EFS.dTomato vector (28) were cloned. For the design of the sgRNAs, the CRISPR/Cas9 target predictor CCTop (29, 30) was used with the settings target site length of 20 amino acids, maximal number of mismatches of three, a core length of 10, and custom overhangs (forward: CACCG and reverse: AAAC). We excluded sgRNAs with predicted off-target effects with less than three mismatches (Supplementary Materials). One microliter of the sgRNA-encoding oligonucleotides (MWG Eurofins, 100 µM) was phosphorylated and annealed in complementary pairs using T4 ligase buffer A (Thermo Fisher Scientific #B69) and T4 PNK (Thermo Fisher Scientific #EK0032) *via* the following incubation steps: incubation: 37°C for 45 minutes, inactivation: 95°C for 150 s, cool down: 0.1 °C/s to 22°C. The SGL40C.EFS.dTomato vector was linearized with BsmBI (NEB #R0580) and 3.1 buffer (NEB #B7203) at 55°C. After 40 minutes, FastAP (Thermo Fisher Scientific #EF0654) was added and samples were incubated at 37°C for another 15 minutes. The 8,178 basepair long vector backbone was isolated and purified from an agarose gel using the NucleoSpin Gel and PCR clean-up kit according to the manufacturer’s instructions (Macherey Nagel #740609.50). Vector and sgRNA inserts (1:300 dilution) were ligated using T4 DNA ligase buffer A (Thermo Fisher Scientific #B69) and T4 DNA ligase (Thermo Fisher Scientific # EL0011) for 90 minutes at room temperature and then stored at 4°C. NEB^®^ 5-alpha-competent *E. coli* were transformed with the ligated vectors. For the transduction of Huh-7.5/F-Luc/Cas9 target cells, lentiviral pseudoparticles were produced. For this, HEK 293T cells were seeded on poly-L-lysine-coated (Gibco # A3890401) cell culture dishes and transfected with pCMV_ΔR8.74 (31), pCMV-VSV-G (32), and the respective SGL40C.EFS.dTomato sgRNA vectors using polyethylenimine (PEI) transfection reagent (Polyplus-transfection^®^ #765090) and Opti-MEM (Gibco # 11520386). The transfection efficiency was induced by sodium butyrate addition at 24 hours (Merck KGaA # 8175000100), the supernatant was collected 72 hours post-transfection and passed through a 0.45-µm filter to remove cell debris (VWR International, LLC #514–1271). For the generation of CRISPR/Cas9-mediated knock-out cells, Huh-7.5/F-Luc/Cas9 cells were transduced with the respective lentiviral pseudoparticles and infected with R-Luc expressing HCoV-229E virus 96 hours post-transduction with 3.41 × 10^6^ TCID_50_/mL. Infected cells were incubated at 33°C for 48 hours and lysed in 0.5% triton X100 in PBS. Renilla and Firefly luciferase signals were quantified separately using a plate luminometer (Berthold Technologies GmbH & Co.KG).

### SARS-CoV-2 infection of Calu-3 and primary human airway epithelial cells

For infection with SARS-CoV-2, Calu-3 cells (26) were seeded in 96-well plates 48 hours prior to virus inoculation at a density of 5 × 10^4^ in DMEM (Gibco #41965039) supplemented as described above. Two hours prior to infection, cells were treated with respective compounds in a volume of 150 µL per well at 37°C. Infection with SARS-CoV-2 was conducted with an infectious dose of 68.2 TCID_50_/well for Calu-3 cells, corresponding to 1.36 × 10^−3^ TCID_50_/cell and resulting in a final volume of 200 µL/well. For infection of well-differentiated primary human airway epithelial cells cultured under air-liquid interface conditions, cells were inoculated with 112 FFU/well of SARS-CoV-2 for 1 hour at 37°C in the presence of increasing concentrations of the respective compound. To determine the assay background, heat-inactivated inoculum (70°C, 15 min) was used. Following incubation for 48 hours at 37°C, supernatants were harvested from Calu-3 cells and inactivated by the addition of lysis buffer (Maxwell 16 viral total nucleic acid purification kit, Promega #AS1150) complemented with proteinase K. To analyze the production of progeny SARS-CoV-2 particles in primary human lung epithelial cells, the apical cell layer was washed twice with a total of 200 µL of Hank’s Balanced Salt Solution (HBSS, Gibco; #14175129) and inactivated as described above. Primary human airway epithelial cells were homogenized in a homogenization solution supplemented with 1-thioglycerol and lysed by the addition of lysis buffer (Maxwell 16 LEV simplyRNA cells kit; Promega #AS 1270). Prior to RNA extraction, all samples were heat-inactivated at 70°C for 15 min. For fluorescence imaging, Calu-3 cells were fixed in 10% formalin for 30 min.

### RNA extraction and qRT-PCR analysis

RNA from lysed and heat-inactivated samples was purified according to the instructions of the manufacturer using a Maxwell 16 viral total nucleic acid purification kit (Promega, #AS1150) or a Maxwell 16 LEV simplyRNA cell kit (Promega; #AS1270), respectively. Purified RNA was eluted in 50 µL nuclease-free water and stored at −80°C. Quantitative RT-PCR was performed using a LightCycler 480 machine (Roche, software version 1.5.1.62), primers and probe specific for SARS-CoV-2 RdRP (TibMolBiol; #53–0777-96) and a LightCycler Multiplex RNA Virus Master Kit (Roche; #07083173001) according to the instructions of the manufacturer. Quantification of copy numbers was done using an in-run standard curve. For GAPDH detection, the following primer and probes were used (S-GAPDH, 5′-GAA GGT GAA GGT CGG AGT C-3′; A-GAPDH, 5′- GAA GAT GGT GAT GGG ATT TC-3′; probe: LC640-CAA gCT TCC CgT TCT CAg CCT—BBQ; TibMolBiol). Single measurements for each sample and duplicate measurements for P-3622 are given.

### Fluorescence microscopy and quantitative image analysis

Following infection, cells used for immunofluorescence staining were fixed with 10% formalin for 30 minutes. Immunofluorescence staining was conducted using the SARS-CoV-2 nucleoprotein (N) mouse monoclonal antibody (1:500; Sino Biological #40143-MM05), Alexa-Fluor Plus 488 anti-mouse antibody (1:1,000; Invitrogen #A32723), and DAPI (1:10,000; Invitrogen #D21490). Four images per well were taken at 20× magnification with an EVOS M5000 microscope (Invitrogen, software version 1.3.660.548) for subsequent computational analysis. Image quantification was performed using Cellprofiler 2.2.0 (33). The analysis pipeline was elaborated and optimized using pictures of cells infected with a blinded dilution series of SARS-CoV-2. Rolling-ball background subtraction was performed for both channels using the ImageJ command (34). Given the small cell size, individual cells were segmented based on the Otsu thresholding of the DAPI channel. Note that for six wells one picture out of the four pictures taken was excluded from the quantification because of poor nuclei segmentation, as judged by eye. Infected cells were distinguished based on the SARS-CoV-2 N signal (Alexa-Fluor Plus 488 channel) (Fig. S6A). To distinguish between infected and uninfected cells, a mean intensity threshold was determined as the 99% quantile of the mean cell intensity in the uninfected control wells of the respective plate (Fig. S6B through D). Cells with mean intensity above the threshold were marked as positive and cells below or equal to the threshold were marked as negative (Fig. S5A). The percentage of positive cells per condition was grouped in Python 3.8.4 and plotted against the relative cell density (overall cell count per condition relative to the overall cell count in the infected and DMSO-treated control).

### Lactate dehydrogenase assay

To quantify the cytotoxic effects of compound treatment and/or gene knock-out, the lactate dehydrogenase (LDH) activity was measured with the lactate dehydrogenase-based *in vitro* toxicology assay kit (Sigma-Aldrich #TOX7) according to the manufacturer’s instructions, method 2. In brief, 50 µL of culture supernatant and 100 µL of Lactate Dehydrogenase Assay Mixture were incubated for 20–30 min. The reaction was stopped by the addition of 15 µL of 1 N HCl and light absorption was detected at 690 nm and 490 nm with a BioTek Synergy 2 plate reader (Biotek Instruments). Background values from media alone were subtracted from all samples.

### Culture of primary cells

Isolation and establishment of well-differentiated primary human airway epithelial cells were done as recently described (35). In brief, human tracheobronchial material from explanted human lungs was enzymatically digested for 48 hours at 4°C prior to the isolation of epithelial cells from the lumen. Cells were seeded on collagen-coated transwells with 0.4 µm pore size, polyester membrane inserts (Corning; #3470) and cultured at 37°C and 5% CO_2_. When confluence was reached, cells were washed with HBSS and exposed to the air. Medium change of the basolateral compartment was performed every other day and cells were washed once weekly with HBSS to remove mucus. Two hours prior to infection, the cells were apically washed with HBSS twice and the basolateral medium was exchanged for medium containing indicated concentrations of the respective drugs. Virus inoculation was performed for 1 hour at 37°C from apical in the presence of drugs prior to removal of inoculum. Apical washing with HBSS and further culturing of cells as ALI cultures occurred at 37°C and 5% CO_2_. Lung transplantations were conducted at Hannover Medical School (MHH) in accordance with good clinical and ethical practice. All patients (>18 years of age) gave informed consent for tissue donation and the project was approved by the local ethics committee at MHH (ethics vote 3346/2016). Cells were tested negative for contamination with mycoplasma (Eurofins).

### Pathway analysis

Pathway analysis and acquisition of metadata describing the ReFRAME drug repurposing collection was mainly conducted based on the information provided by Scripps Research Institute (https://reframedb.org) (21) and the Ingenuity Pathway Analysis software suite (IPA, QIAGEN Inc., https://www.qiagenbio-informatics.com/products/ingenuity-pathway-analysis). To complement this information, the following sources were used: DrugBank (https://go.drugbank.com/) (36), PubChem (37), Guide to PHARMACOLOGY (38), and NCBI PubMed (39). Data were processed *via* Microsoft Excel (Version 2016), GraphPad Prism (Version 8), and Adobe Illustrator. All data were acquired in December 2020, and data for previous screening results (reframedb.org) were updated in October 2022.

## RESULTS

### Profiling of the ReFRAME drug repurposing collection against HCoV-229E, a human-tropic alphacoronavirus

**Fig 2:**
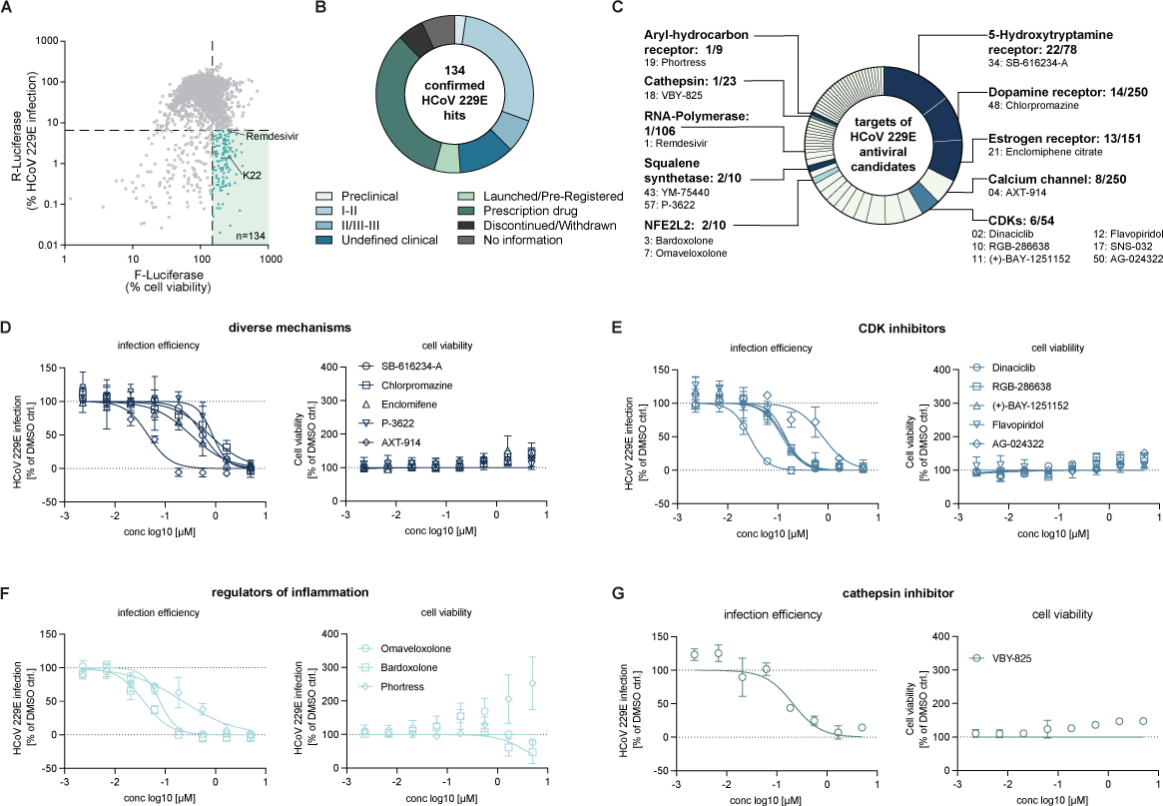
Results of ReFRAME library screening against the Alphacoronavirus HCoV-229E. (**A**) Results of the dual luciferase-based HCoV-229E/R-Luc screening of the ReFRAME library in Huh-7.5/F-Luc cells. *Renilla* luciferase (R-Luc) activity is proportional to virus infection, whereas firefly luciferase (F-Luc) activity corresponds to cell numbers and viability. R-Luc and F-Luc values detected in DMSO-treated, HCoV-229E-infected cells were set to 100% and corrected for background values in the case of R-Luc. The data were generated in one experiment (*n* = 1) with single luciferase measurements. (**B**) Stage of development of confirmed HCoV-229E hit compounds. (**C**) Fifty-eight distinct host targets are annotated to these hits. The number before the slash indicates the number of antiviral hits with this target annotation, the number after the slash is equivalent to the total number of molecules within this target category in the ReFRAME library. The number preceding the compound name indicates the IC_50_ rank (see also Fig. S2 and Table S1). (**D–G**) Dose-dependent antiviral activity against HCoV-229E of the compound categories (**D**) compounds with diverse mechanisms, (**E**) CDK inhibitors, (**F**) regulators of inflammation, and (**G**) cathepsin inhibitors. One experiment with means of two independent luciferase measurements normalized to DMSO control is presented (*n* = 1).

To identify small molecules with broad-spectrum anti-coronavirus activity, we used a sequential screening strategy (Fig. 1B). First, we screened the comprehensive ReFRAME drug library using an HCoV-229E *Renilla* luciferase reporter virus (HCoV-229E/R-Luc) to infect Huh-7.5/F-Luc cells, which are highly permissive for HCoV-229E infection (Fig. S1). These cells are engineered to express a firefly luciferase reporter gene, which permits the assessment of cell viability and infection efficiency in a dual luciferase reporter assay. Dual luciferase reporter assays for high-throughput drug screening have been described previously (40). In total, approximately 12,000 molecules were analyzed. Using stringent inclusion criteria of ≤6.5% virus infection and >150% cell viability, we identified a total of 134 primary hits (Fig. 2A). We chose a high cell viability threshold because HCoV-229E/R-Luc infection of these cells is lytic so that non-toxic, antiviral molecules enhance cell survival. We next confirmed all 134 candidates using the same cellular system and an 8-step dose series for each molecule, thus deducing dose-activity relationships and IC_50_ values for each candidate (Fig. S2). These 134 confirmed primary hits correspond to an overall hit rate of 1%. According to the ReFRAME database, 115 of these hits were screened against SARS-CoV-2 before, and 45 out of 134 confirmed HCoV-229E hits had emerged as primary hits in a SARS-CoV-2-screening campaign (3) and as yet unpublished studies, making them potential broad-spectrum coronavirus antiviral candidates (Table S1). More than 50% of the 134 HCoV-229E hits are either in phase 2 to 3 clinical development, launched, or available as prescription drugs (Fig. 2B). Metadata of these molecules implicate at least 58 distinct drug target categories, thereby defining a landscape of potential host targets for antiviral therapy against an alphacoronavirus (Fig. 2C; Table S1; Fig. S3).

The most frequent target categories of our confirmed HCoV-229E hits were the 5-hydroxytryptamine receptor (22 compounds; e.g. SB-616234-A), the dopamine receptor (14 compounds; e.g., Chlorpromazine), and the estrogen receptor (13 compounds; e.g., Enclomifene) (Fig. 2C and D). For eight molecules, calcium channels are listed as targets (e.g., AXT-914; Fig. 2C and D). Notably, six of 54 cyclin-dependent kinase inhibitors (CDKis) included in the ReFRAME library (April 2021) were found to have antiviral activity (Fig. 2C and E). Five out of six ranked among the top 50 most active HCoV-229E hits, highlighting the relevance of these kinases for HCoV-229E infection in this cellular system (Fig. 2C and E). Dinaciclib, a phase III CDKi, exhibited an IC_50_ of 27.3 nM making it the second most potent compound in the screen, only surpassed by Remdesivir (IC_50_, 15.7 nM). We also found three compounds, which activate cellular transcription factors: one of them is Bardoxolone (ranked at position 3; Fig. 2C and F) which was recently evaluated in a clinical trial for the treatment of hospitalized COVID-19 patients (BARCONA, ClinicalTrials.gov Identifier: NCT04494646). The second one is Omaveloxolone (ranked at position 7; Fig. 2C and F). Both compounds activate the NFE2-like bZIP transcription factor 2 (NFE2L2 also known as NRF2), which regulates genes involved in oxidative stress responses thus protecting cells from oxidative damage. Moreover, Phortress, an activating ligand of the aryl-hydrocarbon receptor (AHR) transcription factor (41–43), which modulates inflammatory processes, also inhibited HCoV-229E infection with an IC_50_ of 230 nM (Fig. 2C and F). Finally, we also found an antiviral activity of VBY-825, a cathepsin inhibitor (Fig. 2C and G). Collectively, these results highlighted numerous small molecules with potent activity against HCoV-229E. These compounds target a wide and diverse range of host proteins. Thus, their antiviral activity may be due to modulation of host targets.

### Functional relevance of host cell drug targets for HCoV-229E infection

**Fig 3:**
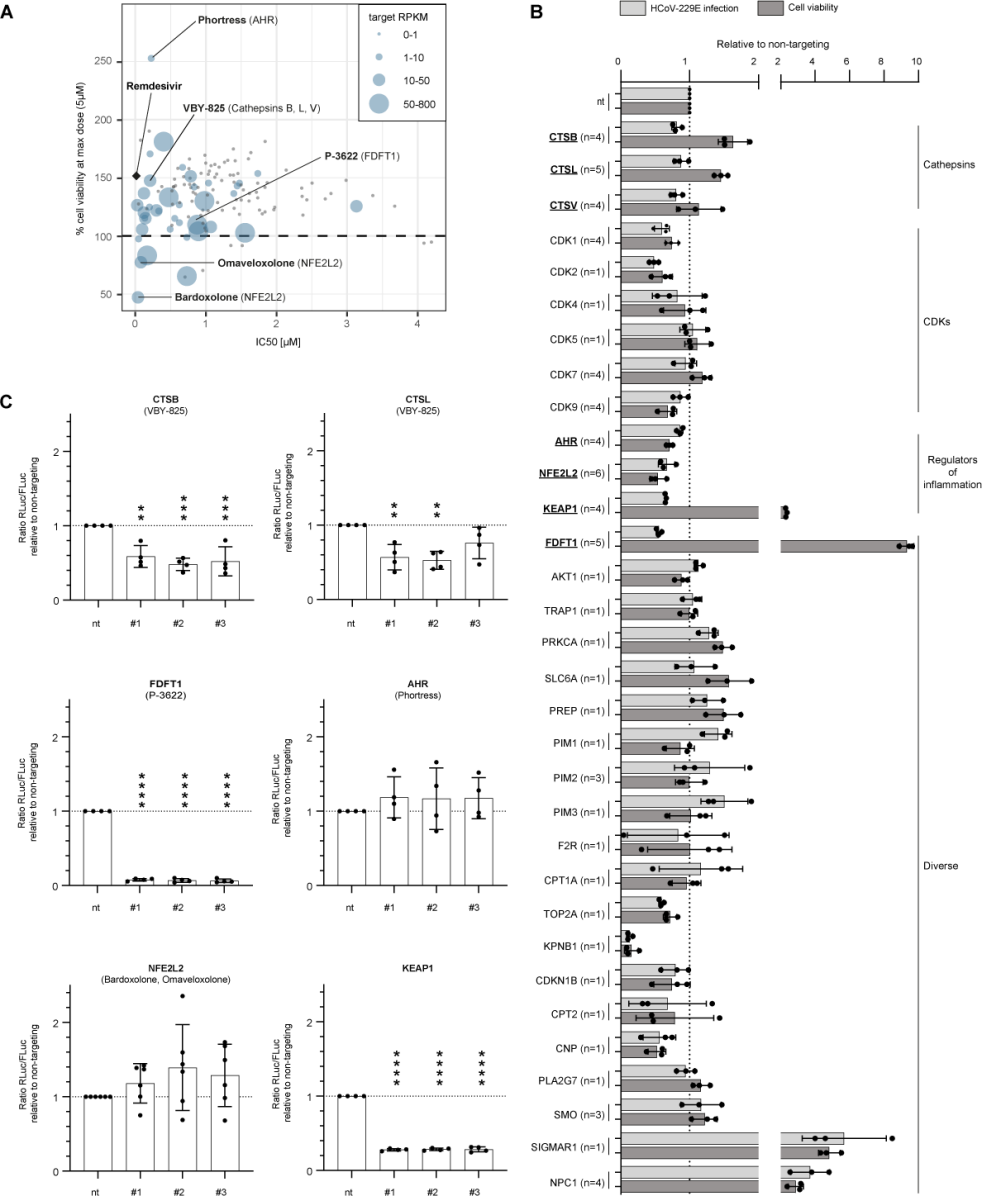
CRISPR/Cas9 screening for potential HCoV-229E host factors. (**A**) Targets for CRISPR-Cas9-mediated knock-outs were selected based on the effect of their corresponding compound on cell viability (≥100% at a concentration of 5 µM) in uninfected Huh-7.5 cells, antiviral effect on HCoV-229E (IC_50_), and RNA expression in Huh-7.5 cells (RPKM ≥1) (*n* = 1). (**B**) Effect of CRISPR/Cas9-mediated knock-outs for 32 putative host targets of a selected number of our hit compounds on HCoV-229E infection (R-Luc, light gray) and cell viability (F-Luc, dark gray). Cells were infected with hCoV-229E for 48 h. The data were normalized to the non-targeting control (nt). The bars represent the mean of the data acquired with three sgRNA per host factor. Each dot represents the mean of all data acquired using one sgRNA with between one and six biological replicates for each sgRNA (1 ≤ n ≤ 6) as indicated after each host factor name. Each biological replicate was composed of three technical replicates. (**C**) Ratio of HCoV-229E infection (R-Luc) to cell viability (F-Luc) normalized to the non-targeting control with data from (**B**). The mean (bar) as well as the individual values (dots) from four to six independent experiments (4 ≤ n ≤ 6) are shown for each sgRNA (#1-#3). One-way ANOVA, Dunnett’s multiple comparisons test, only significant differences compared to the non-targeting control are indicated by asterisks, *P < 0.05, **P < 0.01, ***P < 0.001, ****P < 0.0001.

We reasoned that whether these compounds inhibit HCoV-229E by acting on a cellular target, then genetic inactivation of this target should also modulate HCoV-229E infection. To test this, we conducted a CRISPR/Cas9 knock-out screen focused on the host targets addressed by the 134 hit compounds. Of the 58 host targets, 38 possessed a normalized expression score (reads per kilobase per million reads mapped: RPKM) greater than 1 in our Huh-7.5 screening cell line (Fig. 3A; Table S1) indicating mRNA expression above background. To narrow down gene candidates for a CRISPR/Cas9 screen, we prepared a scatter plot, which relates the cell viability (Y-axis) at the highest compound screening dose (5 µM) to the IC_50_ value (X-axis) of the given molecule and to the mRNA expression level of the given host target modulated by the drug (ball size) (Fig. 3A). Taking these criteria into account allowed us to select candidates which were (i) expressed in the screening cell line, whose blockade was (ii) antiviral and (iii) was not cytotoxic. We selected 32 host targets representing various cathepsins, CDKs, regulators of inflammation, and diverse additional target categories (Fig. 3B). We created Huh-7.5/F-Luc cells stably expressing a Cas9 transgene (Huh-7.5/F-Luc/Cas9) using lentiviral gene transfer and transduced these cells to express the three independent single guide RNAs for each target. Effects of sgRNA expression on cell viability are shown in the supplementary material (Fig. S4). Subsequently, we infected the cells with the HCoV-229E/R-Luc virus and measured F-Luc and R-Luc activity to determine the effects on cell viability and infection efficiency. Transfer of three independent single guide RNAs targeting cathepsin B and L (CTSB, CTSL), respectively, each downregulated HCoV-229E infection and enhanced cell viability (Fig. 3B). This suggested that knock-out of these cathepsins downregulated infection and, in turn, increased cell survival. To validate this observation, we repeated the experiment several times. We plotted the R-Luc activity (infection efficiency) relative to the measured cell viability (F-Luc) (Fig. 3C) and noted a significant downregulation of infection efficiency relative to cell viability for all three CTSB single guide RNAs and two of three CTSL single guide RNAs (Fig. 3C). Collectively, these results confirmed the relevance of CTSB and CTSL for HCoV-229E infection and indicated that VBY-825 likely inhibits HCoV-229E by blockade of the enzymatic activity of cellular cathepsins.

Our screen also revealed that knock-out of sigma non-opioid intracellular receptor 1 (SIGMAR1) and NPC intracellular cholesterol transporter 1 (NPC1) enhanced cell survival and HCoV-229E infection, whereas knock-out of karyopherin subunit beta 1 (KPNB1) strongly downregulated both infection and cell viability (Fig. 3B). Because we were more interested in antiviral and cytoprotective effects, we did not follow up these findings. Therefore, we rather selected AHR, and NFE2L2, the transcription factors activated by the strong antiviral drug candidates Phortress and Bardoxolone/Omaveloxlone, respectively, for further investigation although the effects in the knock-out experiment were not as strong as the aforementioned. We also included kelch-like ECH-associated protein 1 (KEAP1) in our further analysis because this protein binds to NFE2L2 in the cytosol keeping it in its inactive state. Finally, we focused on farnesyl-diphosphate farnesyltransferase 1 (FDFT1) for additional experiments. In total, four to six biological repetitions confirmed a highly significant downregulation of HCoV-229E infection relative to cell viability for all three FDFT1 and KEAP1 single guide RNAs (Fig. 3C). The knock-out of FDFT1 reduced the release of LDH confirming with an alternative assay that knock-out of this host target reduces viral replication and increases cell survival (Fig. 3B and C, and Fig. S5). The effects of AHR and NFE2L2 knock-out were not statistically significant (Fig. 3C). However, we observed a trend toward enhanced HCov-229E infection relative to cell viability which is in line with the notion that the activators of AHR and NFE2L2 were antiviral.

**Fig 4:**
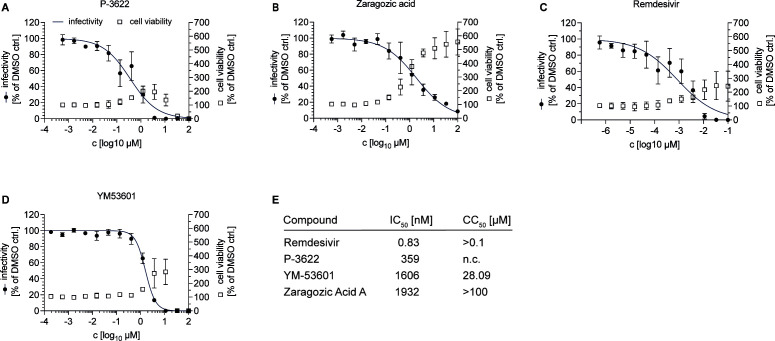
Dose-dependent antiviral activity against HCoV-229E infection of squalene synthases-targeting drugs. (**A-E**) Twelve-step dose-titration of compounds targeting squalene synthases on Huh-7.5/F-Luc/Cas9 cells. Serial 1:3 dilutions of compounds starting at 0.1 µM. The means of three independent experiments with two technical replicates, each, normalized to the DMSO controls are shown (*n* = 3). Dose-titration of (**A**) P-3622, (**B**) Zaragozic acid, (**C**) Remdesivir, (**D**) YM53601. (**E**) IC_50_ (nM) and CC_50_ (µM) values of the titrated drugs using non-linear curve fit calculations shown in A–E (GraphPad Prism 9).

To further corroborate that FDFT1 is a druggable host target and to exclude library artifacts, we re-synthesized the two stereo-isomers of P-3622 and ordered two additional FDFT1 inhibitors [Zaragozic acid A (44) and YM-53601 (45)]. All four compounds dose dependently inhibited HCoV-229E infection (Fig. 4A through F) providing further support for the conclusion that FDFT1 is a druggable HCoV-229E host dependency factor.

### Activity of HCoV-229E hits against SARS-CoV-2 infection of Calu-3 cells

**Fig 5:**
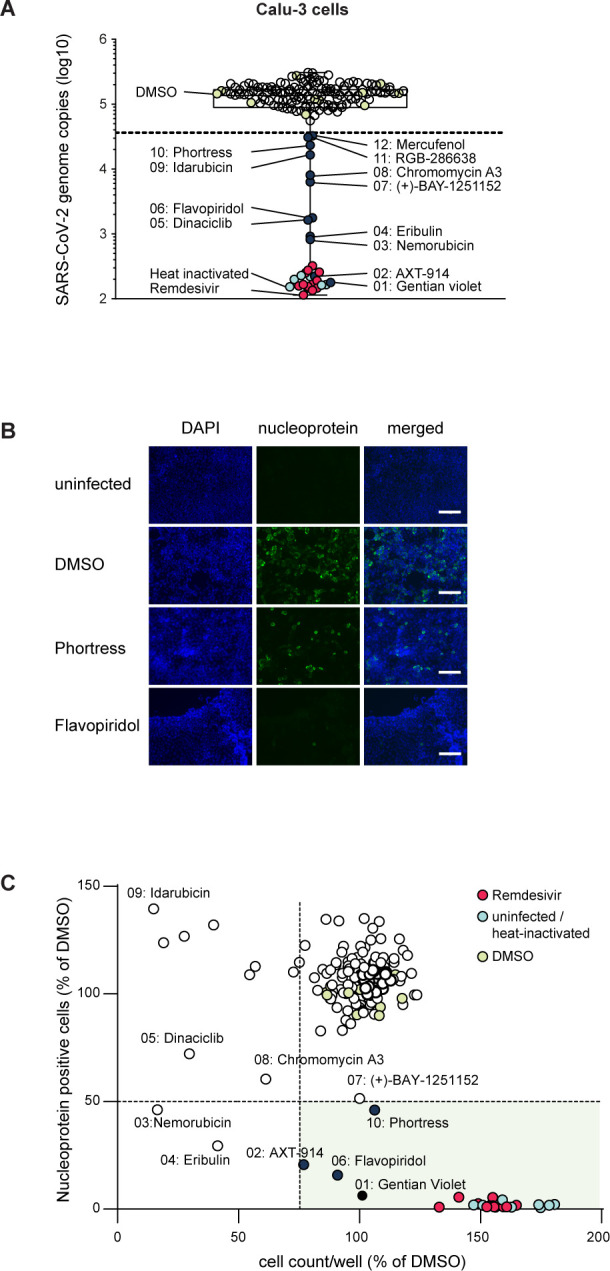
Counter-screening of confirmed HCoV-229E hits with SARS-CoV-2. Calu-3 cells were infected with SARS-CoV-2 (strain SARS-CoV-2/München-1.2/2020/984, **P4**) in the presence or absence of the indicated compounds (final concentration 5 µM, one technical replicate). (**A**) Infection efficiency was analyzed 48 hours post-inoculation by quantification of viral genome equivalents in the culture fluid of cells. qRT-PCR measurements were performed in duplicate. Culture fluid of cells inoculated with heat-inactivated SARS-CoV-2 served as background control (light blue circles). Compounds that reduced virus load more than two standard deviations below the mean value of the DMSO control were recognized as candidate antivirals. (**B**) Immunofluorescence analysis of infected cells. DNA was stained with DAPI (blue), and SARS-CoV-2 nucleoprotein with a monoclonal antibody (green) (scale bar 125 µm). (**C**) Imaging-based quantification of total cell numbers and infected cell numbers. Images were automatically quantified as outlined in the methods section. The relative number of infected cells (N protein expressing cells) is correlated against the number of total cells. Means of four images per well for one experiment (*n* = 1) are presented. DMSO and heat-inactivated virus controls are depicted with light green and light blue circles, respectively. Data from Remdesivir-treated cells are depicted with red circles. Validated broad-spectrum coronavirus antivirals meeting our inclusion criteria are shown with dark blue circles. The inclusion criterion was a reduction of virus-infected cell numbers by more than 50% and a residual total cell number of more than 75% of DMSO solvent control. The data were generated in one experiment due to the limited availability of compounds.

In parallel (Fig. 1B), we determined the potency of the 134 HCoV-229E hits against SARS-CoV-2 (Fig. 5). To this end, we infected the Calu-3 human lung cell line with SARS-CoV-2 (strain SARS-CoV-2/München-1.2/2020/984, p4) (46) in the presence of the HCoV-229E hit substances (screening dose 5 µM). We measured infection by quantification of viral genome equivalents in the culture fluid (Fig. 5A) and by the analysis of total cell and infected cell numbers based on DAPI staining and nucleoprotein immunofluorescence (IF) respectively (Fig. 5B and C). Total cell numbers and frequencies of virus-infected cells were determined in four independent images per well using an automated image processing workflow (Fig. S6 and methods). Besides Remdesivir, 12 compounds reduced the virus load in the culture fluid by at least two standard deviations below the mean value of the DMSO solvent control (Fig. 5A). Among these 12 candidates, Phortress, Flavopiridol, a CDK inhibitor, AXT-914, a calcium-sensing receptor inhibitor, and Gentian violet, an antiseptic dye used for topical treatments of various dermatological conditions, reduced infected cell numbers by more than 50% without reducing total cell numbers to less than 75% (Fig. 5C). Three additional CDKis (Dinaciclib, RGB-286638, (+)-BAY-1251152), and the other candidates, reduced virus load, but did not meet our stringent confirmation criteria based on intracellular N protein staining and cell viability (<50% infected cell, >75% total cells).

### Confirmation of broad-spectrum anti-coronavirus drug candidates in Calu-3 cells and in well-differentiated human airway epithelial air-liquid interface cultures

**Fig 6:**
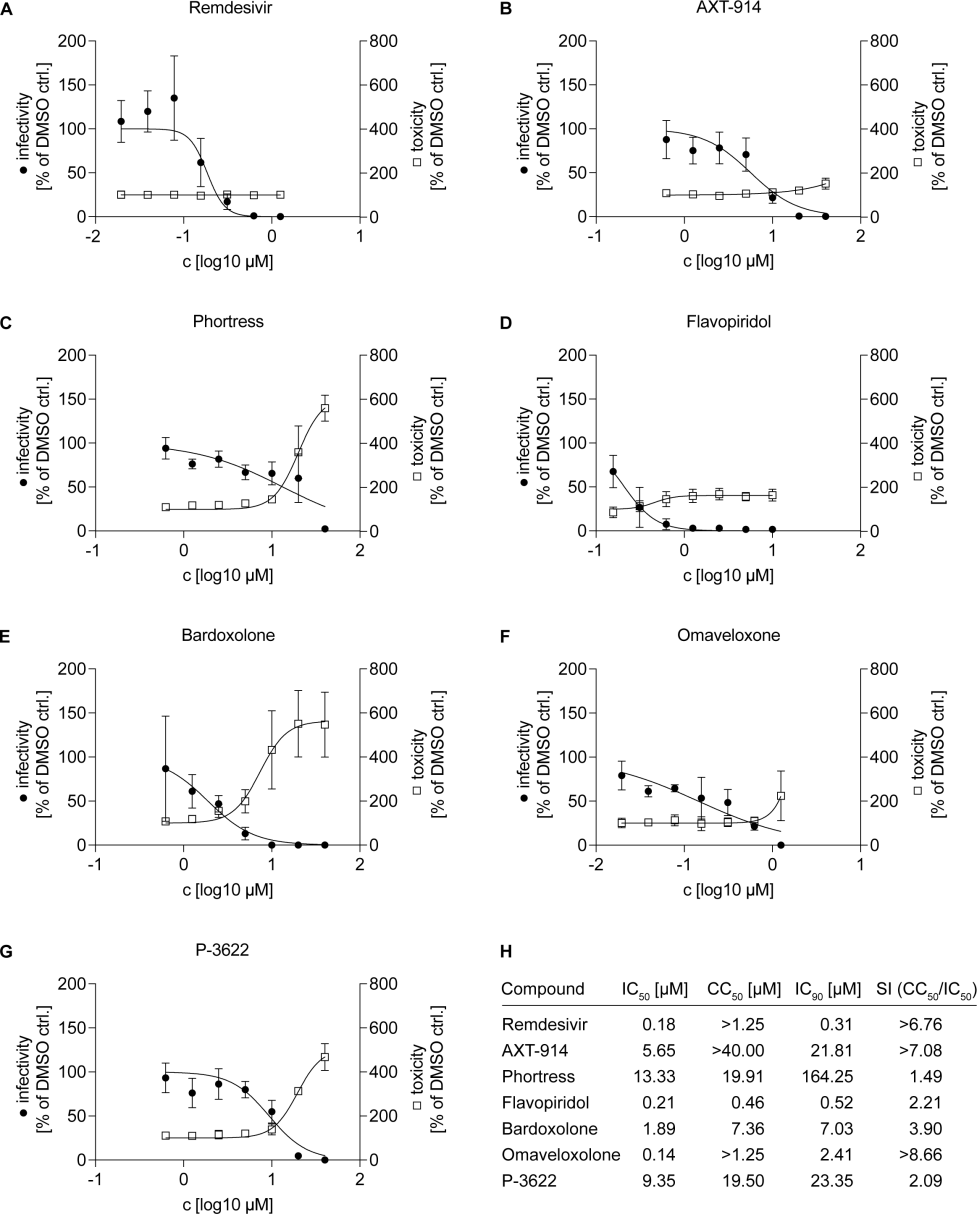
Dose titration of candidates with broad-spectrum coronavirus activity in Calu-3 cells. (**A-G**) Calu-3 cells were seeded 48 hours prior to infection with SARS-CoV-2 and were pre-treated with different doses of the candidate antivirals selected from previous screenings 2 hours before infection. Forty-eight hours post-infection viral genome copies in the supernatant were quantified *via* qRT-PCR. Cytotoxicity was quantified *via* LDH assay using the supernatant of drug-treated, non-infected cells 48 hours after the start of drug treatment. Both infectivity (black dots) and cytotoxicity (clear squares) were normalized to the DMSO control. Mean values of three independent experiments (*n* = 3) with two technical replicates each are shown with a non-linear curve fit including constraints to bottom = 0% and top = 100% (infectivity) and bottom = 100% (cytotoxicity). (**H**) IC_50_, CC_50_, IC_90_, and the specificity index (SI) for each compound calculated from the curve fits shown in (**A-G**).

**Fig 7:**
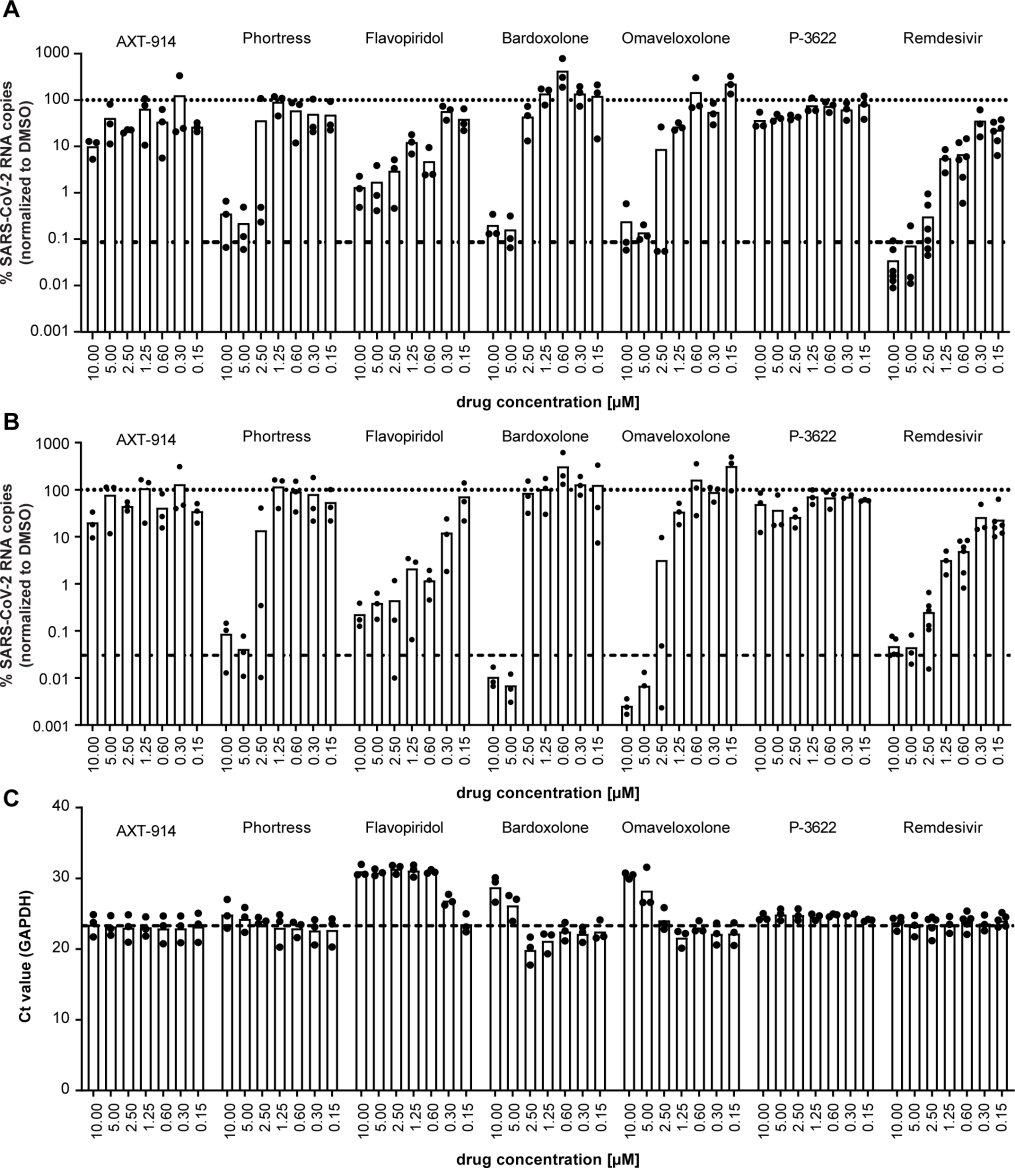
Validation of candidates with broad-spectrum coronavirus activity in well-differentiated primary human lung cells. Primary cells were differentiated and grown as pseudostratified epithelium under air-liquid interface culture conditions. Cells were pre-treated for 2 hours from the basolateral compartment with twofold serial dilutions of indicated compounds starting at 10 µM prior to inoculation with SARS-CoV-2 in the presence of compounds (apical and basal treatment). Virus inoculum was removed 1 hour post-infection and cells were washed once with HBSS and further cultured under air-liquid interface conditions. The drugs in the basolateral compartment were present throughout the experiment. Seventy-two hours post-infection, the new progeny viruses were collected by HBSS washes of the apical compartment prior to lysis of the cells. The antiviral activity of the respective compounds was determined by qRT-PCR analyses of purified RNA from culture fluids (**A**) and cell extracts (**B**). (**C**) Cell viability was assessed by quantification of GAPDH mRNA. Mean values (bar) and individual values (points) of three to six donors are given (*n* = 3 for AXT-914, Phortress, Flavopiridol, Bardoxolone, Omaveloxolone, P-3622; *n* = 6 for Remdesivir). The data were normalized to the respective DMSO solvent control. Dotted lines indicate DMSO solvent control set to 100% and dashed lines give assay background from cells inoculated with non-replicating, heat-inactivated virus. The qRT-PCR analysis from each sample was performed in one technical replicate for AXT-914, Phortress, Flavopiridol, Bardoxolone, and Omaveloxolone and two technical replicates for P-3622 and Remdesivir.

Next, we aimed to confirm a potentially broad antiviral activity of the most interesting candidates by performing dose titrations on Calu-3 cells and well-differentiated primary human airway epithelial (HAE) cells. First, we measured viral genome copies in the supernatant of SARS-CoV-2 infected Calu-3 cells *via* qRT-PCR and assessed drug-induced cytotoxicity of the compounds by measuring LDH release into the supernatant of drug-treated but non-infected cells (Fig. 6). Data were normalized to the respective solvent control and IC_50_ and CC_50_ values were calculated with no universal upper constraint for the cytotoxicity curve fit. For our control Remdesivir, we determined an IC_50_ value of 0.18 µM and we did not observe an increase in LDH activity in the dose range tested (CC_50_ >1.25 µM). All our candidates also inhibited SARS-CoV-2 infection dose dependently with IC_50_ values ranging between 0.14 µM for Omaveloxolone and 13.3 µM for Phortress. AXT-914 and Omaveloxolone showed the best separation of antiviral activity and toxicity in this assay. For the dose titration on HAE cells, we chose a dose range of 0.15 µM to 10 µM as the previous data suggested distinct cytotoxicity at concentrations > 10 µM for most of our candidates. We determined the number of viral genome copies in apical washes (Fig. 7A) and cellular lysates (Fig. 7B). As a measure for cytotoxicity, we determined the Ct value of a qRT-PCR for GAPDH mRNA from the same cellular lysates, thereby indirectly detecting loss of cells due to cell death (Fig. 7C). We tested all our antiviral candidates on cells obtained from three different human donors and our control Remdesivir on cells from six different donors. Remdesivir as well as our antiviral candidates AXT-914 and Phortress dose dependently inhibited SARS-CoV-2 infection without reducing GAPDH mRNA levels. While P-3622 was well tolerated by HAE cells in the tested dose range, it only showed limited antiviral activity. For Flavopiridol and Bardoxolone, the reduction in viral genome copies could not be separated from an increase in cytotoxicity. However, Omaveloxolone, which shares its target with Bardoxolone, inhibited SARS-CoV-2 infection at 1.25 µM and 2.5 µM while maintaining high cell viability. Collectively, these data showed that Phortress, Omaveloxolone, P-3622, and AXT-914 can inhibit HCoV-229E and SARS-CoV-2 infection *in vitro*. Furthermore, these results suggest that the AHR, NFE2L2, and FDFT1 are druggable host targets for the development of host-targeting antivirals against coronaviruses.

## DISCUSSION

This study identifies molecules contained in the ReFRAME library with antiviral activity against a human pathogenic Alphacoronavirus (HCoV-229E). Combined with a CRISPR/Cas9 screen of the host targets modulated by these compounds and a counter-screening using a SARS-CoV-2 isolate representing the genus *Betacoronavirus*, we suggest compounds with potentially broad-spectrum anti-coronavirus activity and their corresponding host targets. While we did not identify any antiviral compounds potent enough to progress to clinical trials directly, we highlight candidates for further development and novel host-target candidates that might lay the path for the development of novel host-targeting antiviral therapies.

Primary screening of approximately 12,000 library compounds and dose-response titrations identified and confirmed 134 candidates against HCoV-229E associated with implicating 58 different potential host target categories. Molecules with the 5-hydroxytryptamine (i.e., serotonin) receptor as target annotation represented the largest group of hits against HCoV-229E. We identified 22 of 78 molecules included in the ReFRAME library representing 28% of compounds in this class. The second most dominant HCoV-229E target category was the dopamine receptor with 14 hits out of 250 molecules contained in the library. Thus, collectively, 36 of 134 HCoV-229E targeting compounds, equivalent to 26.8%, are categorized as neurotransmitter receptor modulators (target information: reframedb.org, February 2021). While off-target antiviral mechanisms are possible and have been described for Sertraline, which is a 5-hydroxytryptamine receptor inhibitor, but blocks SARS-CoV-2 entry by disturbing the spike-receptor interaction (47), the accumulation of hits in these categories remains striking. Importantly, only one molecule (SB-616234-A) has been described in a previous ReFRAME SARS-CoV-2 screening study (3), and none of them scored in our SARS-CoV-2 counter-screening, suggesting that these compounds preferentially target HCoV-229E. The 5-hydroxytryptamine receptor family comprises seven protein groups, six of which encompass G-protein-coupled receptors (GPCRs), whereas the dopamine receptor family includes proteins categorized in two groups of GPCRs (48). Therefore, GPCR-targeting compounds are heavily enriched in the HCoV-229E antiviral screening. The reason for the HCoV-229E preferential accumulation of GPCR modulators is currently unknown, but likely due to key differences in host factor usage between these viruses. One important difference between HCoV-229E and SARS-CoV-2 is their receptor usage with the former exploiting human alanyl aminopeptidase (ANPEP, also known as hAPN/CD13) and the latter ACE2. Both proteins are plasma membrane resident proteases, but neither is a GPCR. ANPEP is expressed in numerous tissues and hydrolyses proteins involved in different physiological processes (49). Intestinal ANPEP processes peptides originating from gastric proteases, whereas neuronal ANPEP for instance is involved in the proteolysis of neurotransmitters. Consistent with the assumption that differences in cell entry between HCoV-229E and SARS-CoV-2 account for the accumulation of these types of inhibitors in the HCoV-229E screen, we noted a stronger inhibition of VSV-pseudotypes coated with the HCoV-229E spike protein as opposed to the SARS-CoV-2 spike protein (Fig. S7). GPCR-targeting neuroleptic drugs have emerged as cell entry inhibitors of other enveloped viruses before, although these viruses also do not use GPCRs directly for cell entry (50). The mechanisms by which this occurs may differ between these viruses and may range from direct binding to the viral fusion protein to the modulation of membrane curvature and fluidity.

We observed cathepsin dependency of HCoV-229E infection, which has been described to be more relevant for cell-culture-adapted strains as compared to clinical isolates (51). Therefore, it should carefully be considered that the entry process of the HCoV-229E strain used might not represent a natural infection and that certain drugs or host factors relevant to a natural infection might not have been identified.

Next to Remdesivir, the CDKi Dinaciclib emerged as the most potent HCoV-229E inhibitor within the ReFRAME library (IC_50_ 27 nM). We identified five additional CDKis, all of which ranked among the top 50 HCoV-229E screening hits with potential antiviral activity and limited cytotoxicity (Fig. 2E; Fig. S2). Thus, the CDKi category hosts the most potent HCoV-229E inhibitors in the Huh-7.5/F-Luc assay. CDKis were not described as SARS-CoV-2 inhibitors by Riva et al. (3), possibly due to the usage of different host cell lines. However, a recent phosphoproteome survey of SARS-CoV-2 infected cells has revealed a time-structured, SARS-CoV-2-dependent modulation of CDK activity (52). These authors also described several potential CDK phosphorylation sites within viral proteins and they found that Dinaciclib inhibits SARS-CoV-2 infection of Vero E6 and A549/ACE2 cells with nanomolar efficacy (52). Flavopiridol, a phase 3 CDKi, qualified as a hit in both imaging- and qRT-PCR-based assays in Calu-3 cells. RGB-286638, (+)-BAY-1251152, and Dinaciclib also reduced SARS-CoV-2 RNA copy numbers in the culture fluid of infected Calu-3 cells, but they did not meet the more stringent inclusion criteria of the Calu-3 imaging-based analysis (Fig. 5). In the 8-step dose-activity titration of these compounds in Huh-7.5/F-Luc cells, we observed a clear separation between antiviral activity against HCoV-229E and cytotoxicity, suggesting a direct inhibition of virus infection. Using a limited dose range in the human primary lung cells, we were unable to separate antiviral effects from cytotoxic effects. Thus, while CDK inhibition seems broadly active against coronaviruses in some cell lines, cytotoxic effects need to be carefully considered. CDKs are essential regulators of cell cycle progression, and their inhibition can especially impact naturally fast proliferating cells such as in the bone marrow which limits the use of CDKis as therapeutics (53).

Phortress, an antitumor benzothiazole prodrug and AHR activating ligand (41–43), exhibited an antiviral activity against HCoV-229E and SARS-CoV-2. We consistently detected antiviral effects of Phortress in all cellular systems including primary differentiated human lung cells. Moreover, knock-out of AHR slightly increased HCoV-229E infection, suggesting that Phortress inhibits coronavirus infection by activating the host transcription factor. Ligands of the AHR are in clinical development for the treatment of kidney cancer (43) (ClinicalTrials.gov Identifier: NCT04069026). AHR itself is a transcription factor that resides in the cytoplasm complexed with the heat shock protein 90 (Hsp90). Upon ligand binding, AHR disassociates from Hsp90, traffics to the nucleus, and transactivates genes downstream of the xenobiotic response element. AHR-dependent genes include metabolic enzymes such as cytochrome (CYP) P450 1A1, CYP1A2, and CYP1B1 (43). AHR also participates in immune regulatory processes: For instance, constitutive activation of AHR downregulates type I IFN-dependent innate immune responses (54). Notably, Kueck et al. reported that AHR activation in macrophages limits cellular dNTP pools, thereby inhibiting replication of herpes- and retroviruses (HSV-1 and HIV-1, respectively) (55). A recent study provided evidence that coronavirus infection increases the expression of AHR and AHR-dependent genes (56). Furthermore, these authors noted that the CH223191 AHR antagonist decreased HCoV-229E and SARS-CoV-2 infection in Huh-7.5, Calu-3, and Vero cells, respectively. On the other hand, Tanimoto and colleagues reported that AHR-agonists 6-formylindolo(3,2-b) carbazole (FICZ) and omeprazole (OMP) inhibited SARS-CoV-2 infection of Vero cells (57). Furthermore, they provided evidence that FICZ and OMP reduce ACE2 expression through the activation of AHR. The reason for the discrepancy between these two studies showing antiviral effects for either antagonists or agonists of AHR is currently unclear. Major et al. recently showed that active AHR signaling prevents influenza infection-induced barrier disruption in the lung (58). In this present study, we show an antiviral effect of Phortress against SARS-CoV-2 infection of primary human lung cells, thereby extending the evidence that AHR agonists are antiviral. While this activity may be linked to modulation of ACE2 expression, such an effect is unlikely to explain the anti-HCoV-229E effect. Thus, more work is needed to find out, which AHR-dependent processes including inflammatory response (59) are relevant for the inhibition of these viruses by AHR modulators.

Bardoxolone and Omaveloxolone exhibited IC_50_ values of 37 nM and 78 nM against HCoV-229E, ranking them at positions 3 and 7, respectively, in our screening hit list. While they did not meet the inclusion criteria of our SARS-CoV-2 Calu-3 cell counter screen at the single dose of 5 µM due to high cytotoxicity, Omaveloxolone exhibited an antiviral activity against SARS-CoV-2 at lower doses in the dose titration on Calu-3 cells (IC_50_ = 0.14 µM) and on primary human lung cells. Both compounds are semisynthetic derivatives of the triterpene oleanolic acid. They bind covalently to kelch-like ECH-associated protein 1 (KEAP1), which leads to the activation of NFE2L2 (NRF-2) (60, 61). If these drugs inhibited hCoV-229E infection by activation of NFE2L2 signaling, a knock-out of NFE2L2 would be expected to promote viral infection. While we did not observe a statistically significant effect, knock-out of NFE2L2 showed a trend toward enhanced infection, whereas knock-out of the endogenous inhibitor KEAP1 strongly decreased infection. Due to its anti-inflammatory and tissue repair effects, NFE2L2 activation has been recently proposed as a strategy to treat COVID-19 (62), and a clinical trial testing safety, tolerability, and efficacy of Bardoxolone methyl in hospitalized COVID-19 patients was assessed (BARCONA, ClinicalTrials.gov Identifier: NCT04494646). In fact, broad-spectrum antiviral properties of Bardoxolone methyl have been reported for a variety of viruses including dengue virus (DENV), Zika virus (ZIKV), hepatitis B virus (HBV), hepatitis C virus (HCV), and herpes simplex virus 1 (HSV-1) (63–65). Specifically, the NFE2L2-dependent expression of antioxidant genes was found to be suppressed in biopsy samples obtained from COVID-19 patients, and the administration of the primary metabolite prodrugs 4-octyl-itaconate (4-OI) and dimethyl fumarate (DMF), that both activate NFE2L2, had anti-SARS-CoV-2 effects at high concentrations (66). Bardoxolone methyl has progressed to phase 3 clinical trials, mainly as a long-term treatment of kidney diseases. Although its late-stage clinical development faced setbacks due to the occurrence of cardiovascular side effects in the BEACON study (67), the mechanisms and safety biomarkers for such side effects have been identified, and the clinical development of Bardoxolone methyl has been resumed (68). In addition, Omaveloxolone is in late-stage clinical trials for a neurological indication (69).

Repurposed drugs have already been approved for several diseases. Examples include Ritonavir, initially approved to treat HIV infections and now used in combination with Nirmatrelvir to treat SARS-CoV-2 infections (70), and Tenofovir, a nucleotidic HIV reverse transcriptase inhibitor that was later licensed for the treatment of hepatitis B virus infections (71, 72).

Furthermore, other examples highlight the importance of considering host-targeting molecules for the discovery of novel treatment options. For instance, Maraviroc is a C-C chemokine receptor type 5 antagonist that prevents HIV cell entry (73), and Bulevirtide, currently conditionally approved by the European Medicines Agency (EMA), blocks cell entry of hepatitis Delta virus (HDV) by binding to its cellular receptor sodium taurocholate cotransporting polypeptide (NTCP) (74). Another therapeutic currently in a phase III clinical trial for the treatment of HDV infection is Lonafarnib (75). Lonafarnib (Zokinvy) is a farnesyl transferase inhibitor approved for the treatment of Hutchinson-Gilford progeria syndrome (76). Here, we aimed to provide further evidence that repurposing screens are not only suitable for drug repurposing studies and the identification of active compounds but also for the identification of druggable viral host targets—potentially with broad-spectrum relevance for diverse viruses. Using a combination of drugs and selected CRISPR/Cas9 host drug target screening, we confirmed the relevance of CTSB/L proteases for coronavirus infection. In addition, we provide evidence that NFE2L2/KEAP1, FDFT1, and AHR are potentially druggable host targets with relevance for various viral infections. Previous SARS-CoV-2 CRISPR screens had also shown the importance of NFE2L2/KEAP1 (77) and FDFT1 (78). Thus, our work confirms these two studies regarding coronavirus host factor usage. Adding to this, we provide evidence that Omaveloxolone may be more suitable than Bardoxolone because it was better tolerated in primary human lung cells. Furthermore, we show that chemically diverse FDFT1 inhibitors exert antiviral effects. This is of particular interest considering that FDFT1 is important not only for infection by coronaviruses (this present study and (78)) but also the hepatitis C virus (79) and an enterovirus such as encephalomyocarditis virus (EMCV) (78). Considering these viruses belong to three different families of plus-strand RNA viruses, and the success of plus-strand RNA viruses as zoonotic pathogens, our finding should draw further attention to the mechanistic role of FDFT1 for infection including the utility of this host target for broad-spectrum antiviral therapy.

### Conclusions

This screening of the ReFRAME repurposing library identified 134 hit candidates with confirmed activity against the human pathogenic alphacoronavirus HCoV-229E. Combined with a CRISPR/Cas9 screening of host targets and a validation of compounds in a SARS-CoV-2 infection assay in primary human lung cells, we found a potentially druggable host factor dependence of HCoV-229E and SARS-CoV-2 for the transcription factors AHR (Phortress), NFE2L2/KEAP1 (Omaveloxolone) and for FDFT1 enzyme (P-3622).

## SUPPLEMENTAL MATERIAL

Supplemental figures and materials and methods aac.01210-23-s0001.docx Figures S1-S7 and supplemental materials and methods.

Table S1 aac.01210-23-s0002.xlsx List of validated compounds inhibiting HCoV 229E infection of Huh-7.5/F-Luc cells.

ASM does not own the copyrights to Supplemental Material that may be linked to, or accessed through, an article. The authors have granted ASM a non-exclusive, world-wide license to publish the Supplemental Material files. Please contact the corresponding author directly for reuse.
