# Supplementary material for: Repurposing screen identifies novel candidates for broad-spectrum coronavirus antivirals and druggable host targets
Source: Antimicrob Agents Chemother. 2024 Feb 6;68(3):e01210-23. doi: 10.1128/aac.01210-23 (PMC10916382; doi:10.1128/aac.01210-23)
Supplement: Supplemental figures and materials and methods — Figures S1-S7 and supplemental materials and methods. [file aac.01210-23-s0001.docx]

**Supplementary Information for**

Novel candidates for broad-spectrum coronavirus antivirals and druggable host targets

Sibylle Haid^1†^, Alina Matthaei^1†^, Melina Winkler^1†^, Svenja M. Sake^1^, Antonia P. Gunesch^1^, Vanessa Milke^1^, Natalie M. Köhler^1^, Jessica Rückert^2,3^, Gabrielle Vieyres^4,5^, David Kühl^4^, Tu-Trinh Nguyen^6^, Matthias Göhl^3,7^, Lisa Lasswitz^1,8^, Francisco J. Zapatero-Belinchón^1,8^, Graham Brogden^1,8^, Gisa Gerold^1,8,9,10,11^, Bettina Wiegmann^12,13,14^, Ursula Bilitewski^7^, Richard J. P. Brown^15^, Mark Brönstrup^3,7^, Thomas F. Schulz^2,3,11^, and Thomas Pietschmann^1,3,11*^

^1^Institute for Experimental Virology, Twincore - Centre for Experimental and Clinical Infection Research; 30625 Hannover, Germany.

^2^Institute of Virology, Hannover Medical School; 30625 Hannover, Germany.

^3^German Center for Infection Research, Hannover-Braunschweig Site; 30625 Hannover, Germany.

^4^Junior Research Group “Cell Biology of RNA Viruses”, Leibniz Institute of Experimental Virology; 20251 Hamburg, Germany.

^5^Integrative Analysis of Pathogen‑Induced Compartments, Leibniz ScienceCampus InterACt; 20251 Hamburg, Germany.

^6^Calibr, a Division of The Scripps Research Institute; La Jolla, CA 92037, USA.

^7^Helmholtz Centre for Infection Research, 38124 Braunschweig, Germany.

^8^Department of Biochemistry & Research Center for Emerging Infections and Zoonoses (RIZ), University of Veterinary Medicine Hannover; 30559 Hannover, Germany.

^9^Department of Clinical Microbiology, Virology, 901 87 Umeå University; Umeå, Sweden.

^10^Wallenberg Centre for Molecular Medicine (WCMM), 901 87 Umeå University; Umeå, Sweden.

^11^Cluster of Excellence RESIST (EXC 2155), Hannover Medical School; 30625 Hannover, Germany.

^12^Department of Cardiothoracic, Transplantation and Vascular Surgery, Hannover Medical School; 30625 Hannover, Germany.

^13^Lower Saxony Center for Biomedical Engineering, Implant Research and Development, Hannover Medical School; 30625 Hannover, Germany.

^14^BREATH (Biomedical Research in Endstage and Obstructive Lung Disease Hannover), German Center for Lung Research (DZL), Carl-Neuberg Str. 1, 30625 Hannover, Germany

^15^Division of Veterinary Medicine, Paul Ehrlich Institute, 63225 Langen, Germany.

† These authors contributed equally to this work. Author order was determined based on decreasing seniority relative to the date they obtained their PhD degree.

* Corresponding author: Thomas Pietschmann, Institute for Experimental Virology, Twincore - Centre for Experimental and Clinical Infection Research; Feodor-Lynen-Straße 7, 30625 Hannover, Germany, phone: +49-511-220027130, email: thomas.pietschmann@twincore.de

**Email:** thomas.pietschmann@twincore.de

**This PDF file includes:**

Figures S1 to S7

Legends for Dataset table SI

Supplementary Materials and Methods

Supplementary References

**Other supplementary materials for this manuscript include the following:**

Dataset table SI


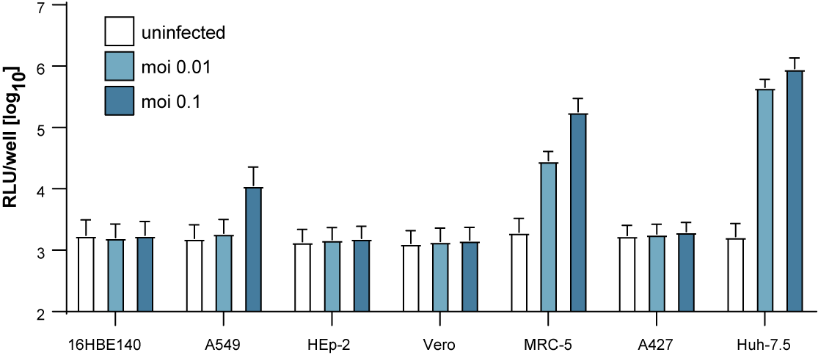


Fig. S1: Screening of human host cell lines for HCoV-229E ReFRAME drug repurposing screen. Given cell lines were inoculated with varying doses of HCoV-229E reporter virus or they were left untreated (uninfected). Fifty-five hours later, cells were lysed and bioluminescence was measured. Mean values and standard deviation of three independent experiments (n=3) each with duplicate wells are shown.


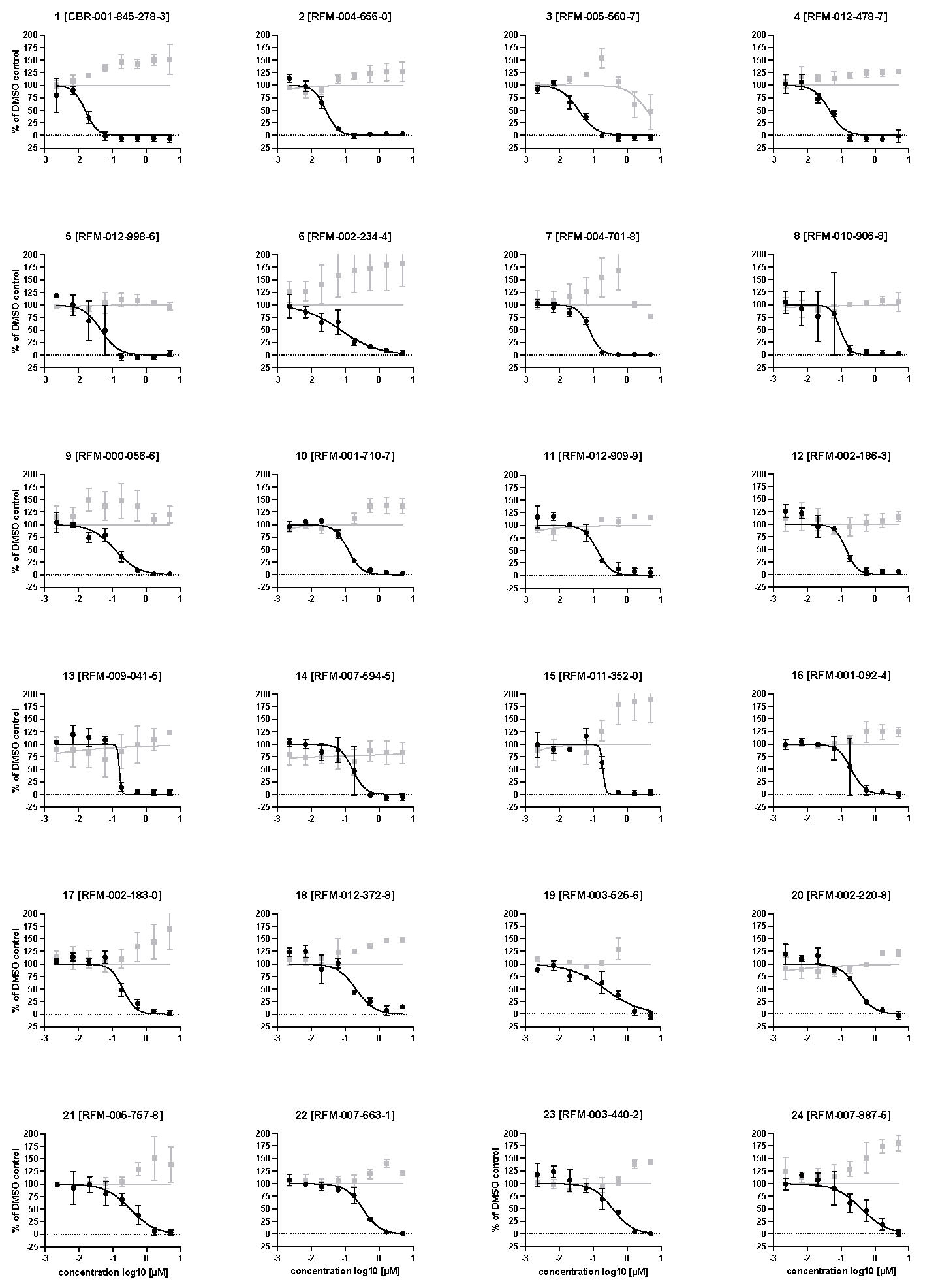

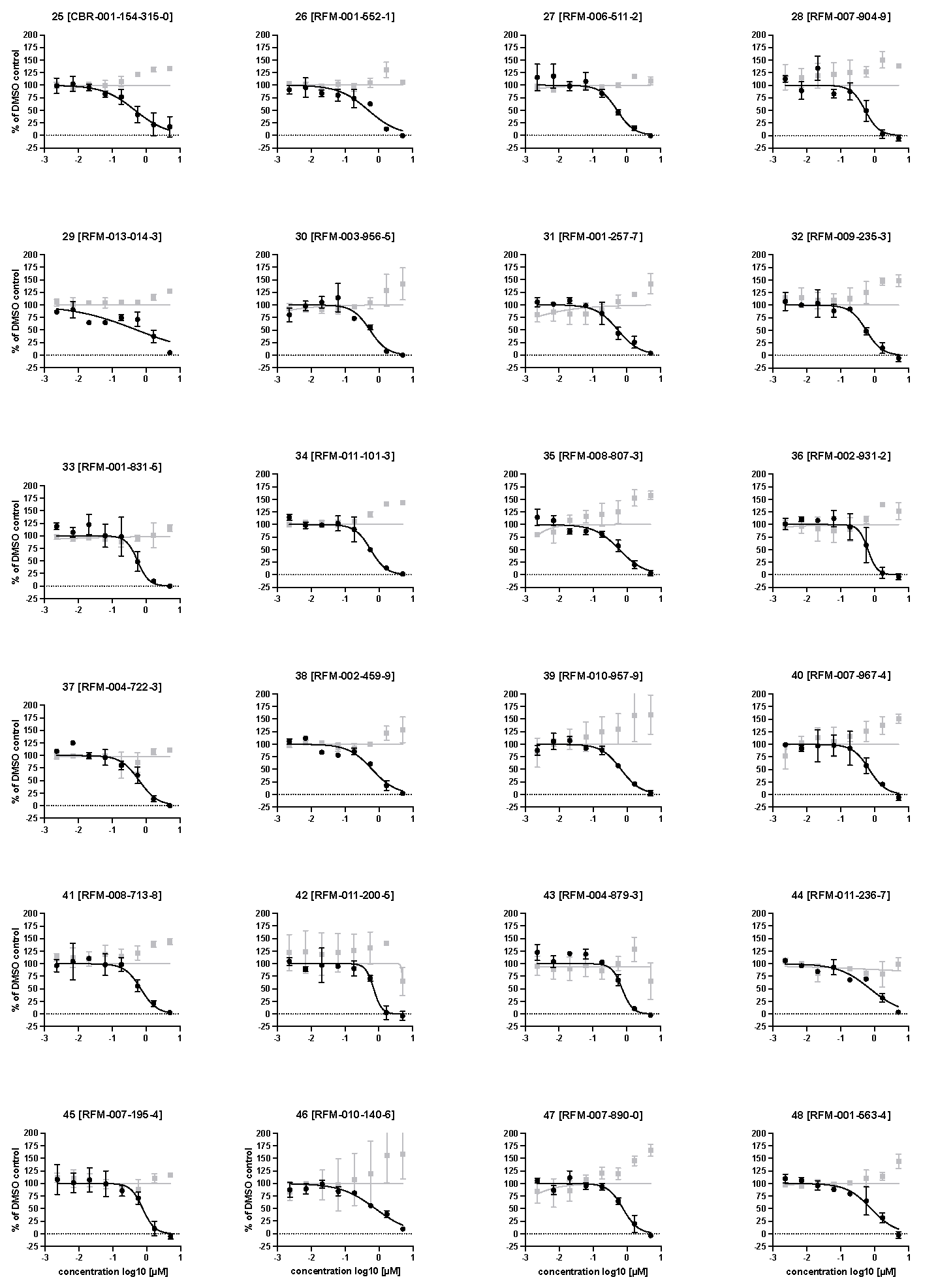

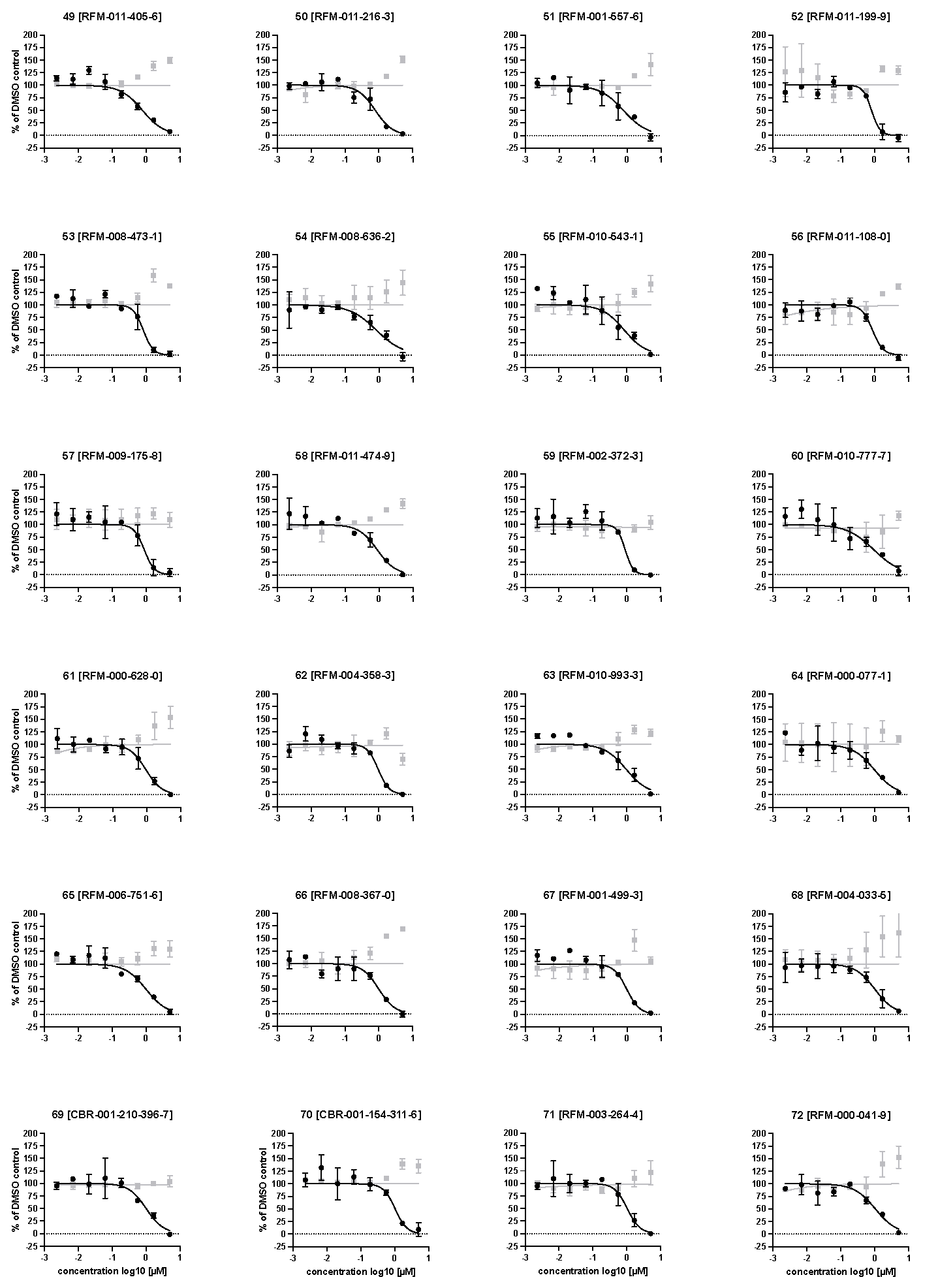

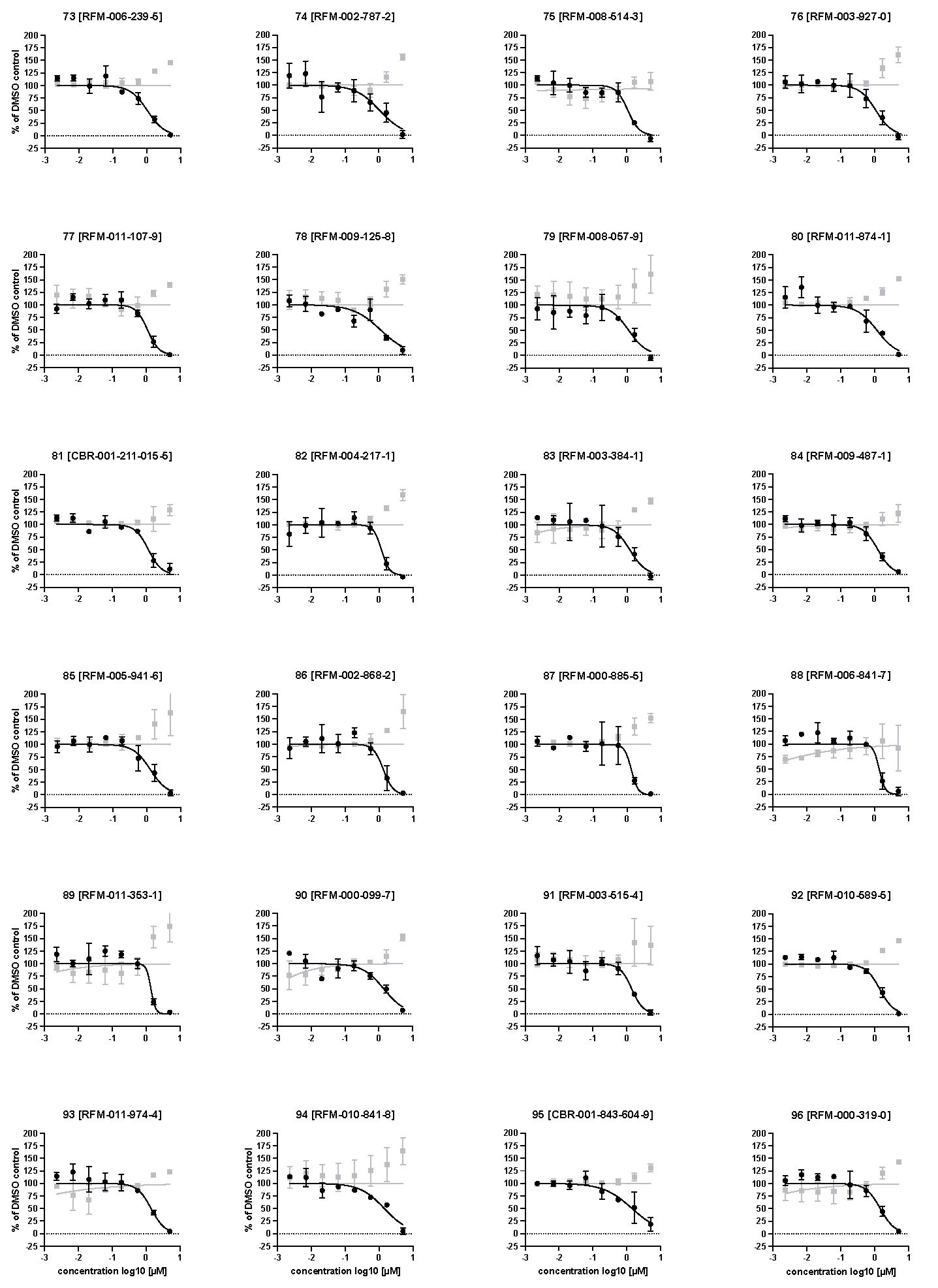

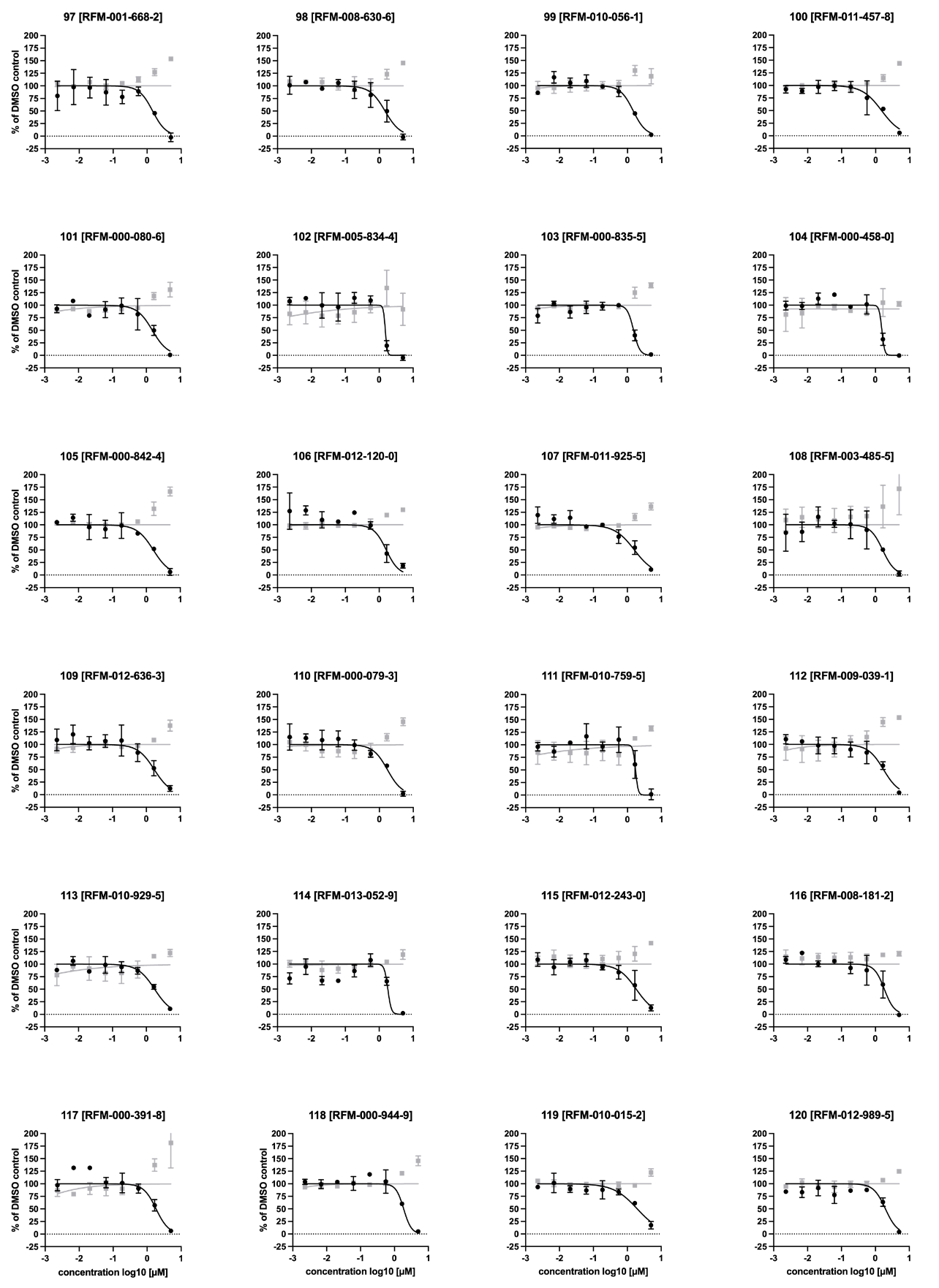

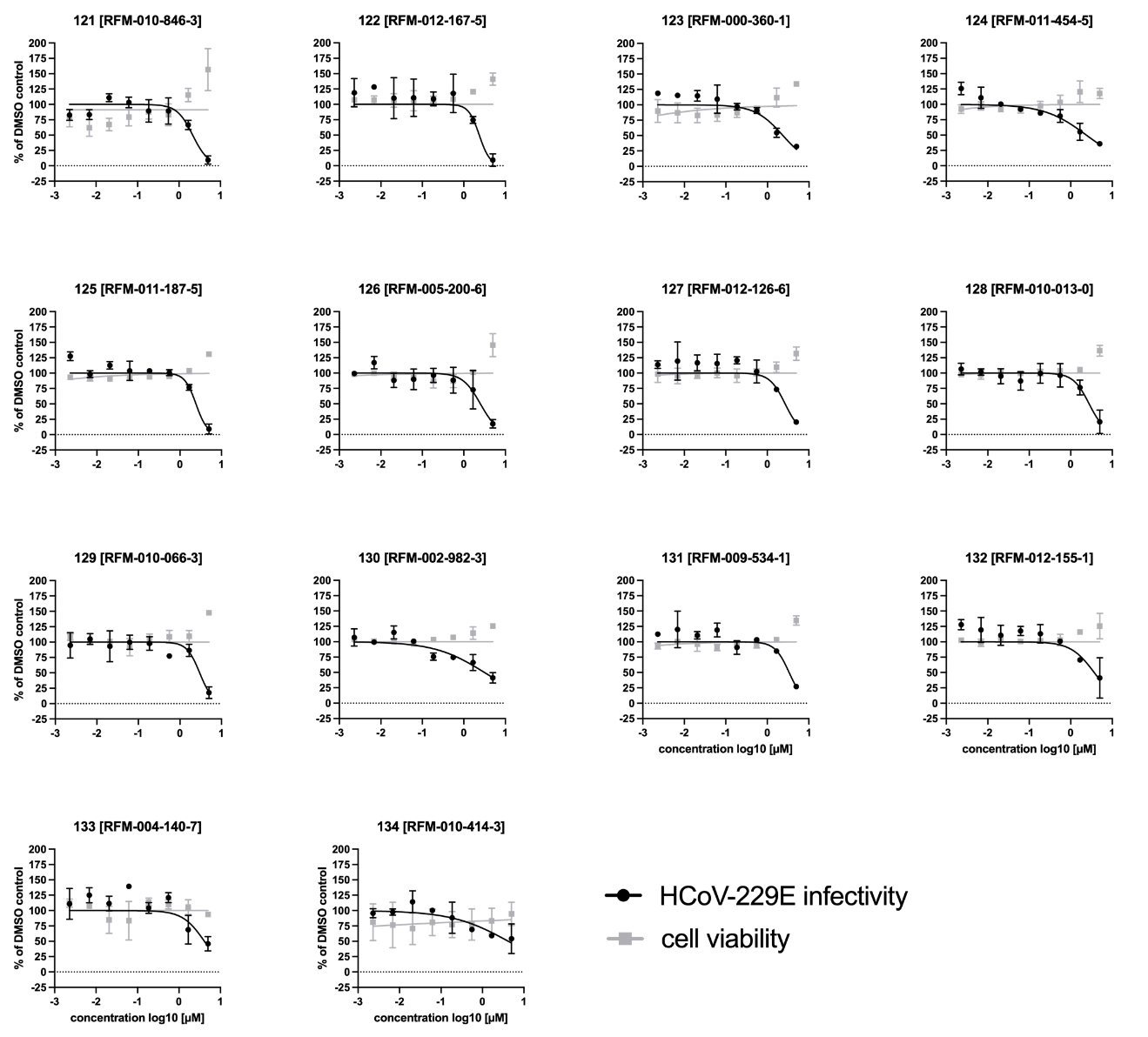


Fig. S2: Dose-response analysis of 134 primary hits from HCoV-229E ReFRAME drug repurposing screening. Serial dilutions of 134 primary hits from the HCoV-229E screening were combined with reporter viruses and used for infection of Huh-7.5/F-Luc cells. Forty-eight hours later cells were lysed, and bioluminescence was determined. *Renilla* luciferase activity corresponds to virus infection efficiency (black curve and dots), whereas firefly luciferase activity reflects cell viability (grey curve and squares). Mean values of duplicate measurements of one experiment (n=1) including standard deviation are depicted and were plotted relative to infections in presence of DMSO. Compounds were rank ordered based on IC_50_ value. IC_50_-rank and ReFRAME identifiers are given on top. Compound details are listed in table SI.


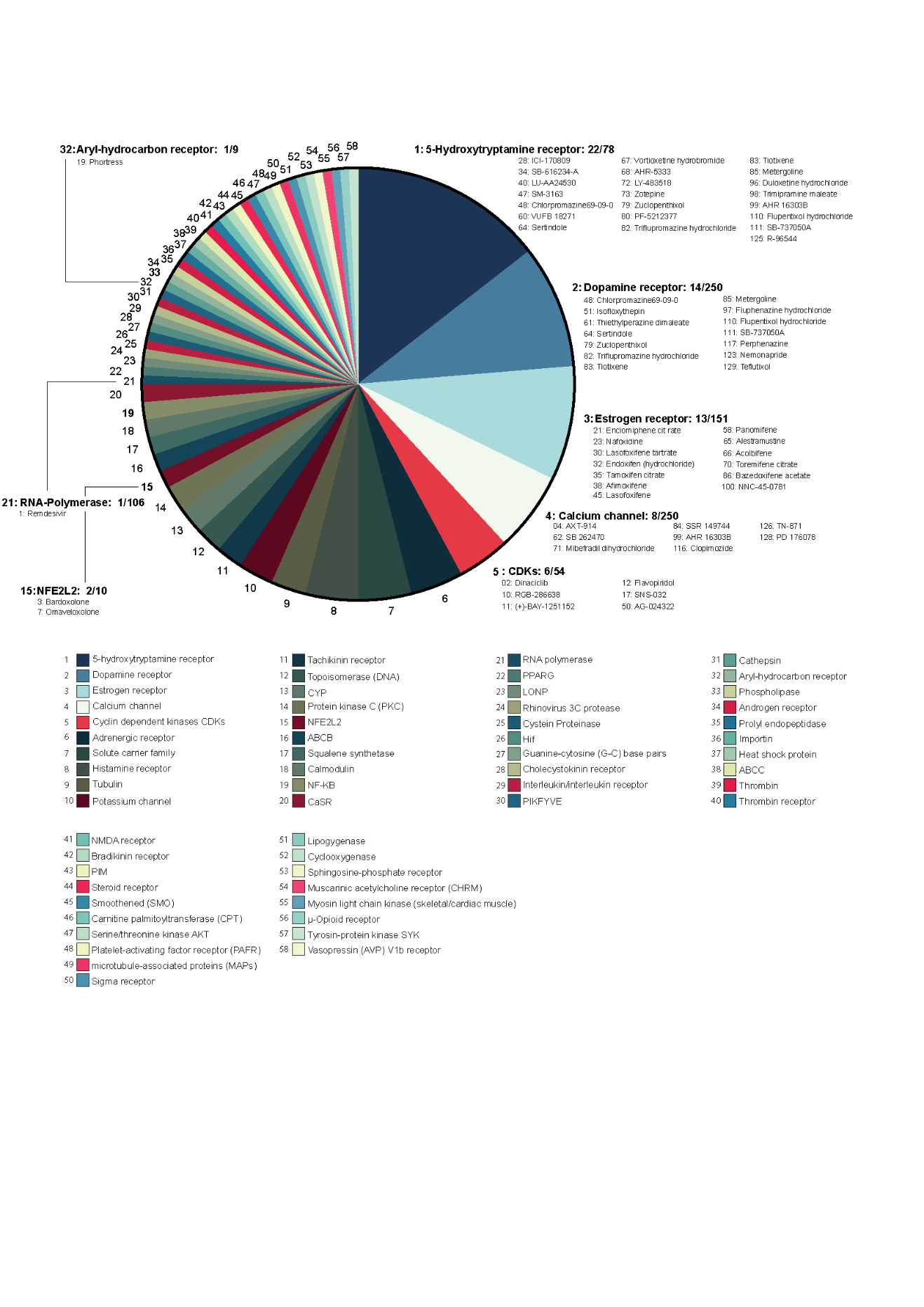


Fig. S3: Complete overview of annotated host targets of 134 confirmed HCoV-229E screening hits. Screening hits were categorized for their specific putative host target. Host target groups were sorted by size. Numbers in front of targets indicate the rank of target in the listing. Numbers before the dashes represent the number of compounds that were detected in our HCoV-229E screen. The number after the dash is equivalent to the total number of compounds belonging to this target group and comprised in the library (reframedb.org database, December 2020). For the five largest groups, as well as for NFE2L2-, RNA-polymerase and AHR-targeting molecules, the respective compounds identified as hits in this screen are named. Numbers in front of the compounds constitute the rank number of the respective compound in our HCoV-229E screen. Rank ordering was conducted based on IC_50_-values.


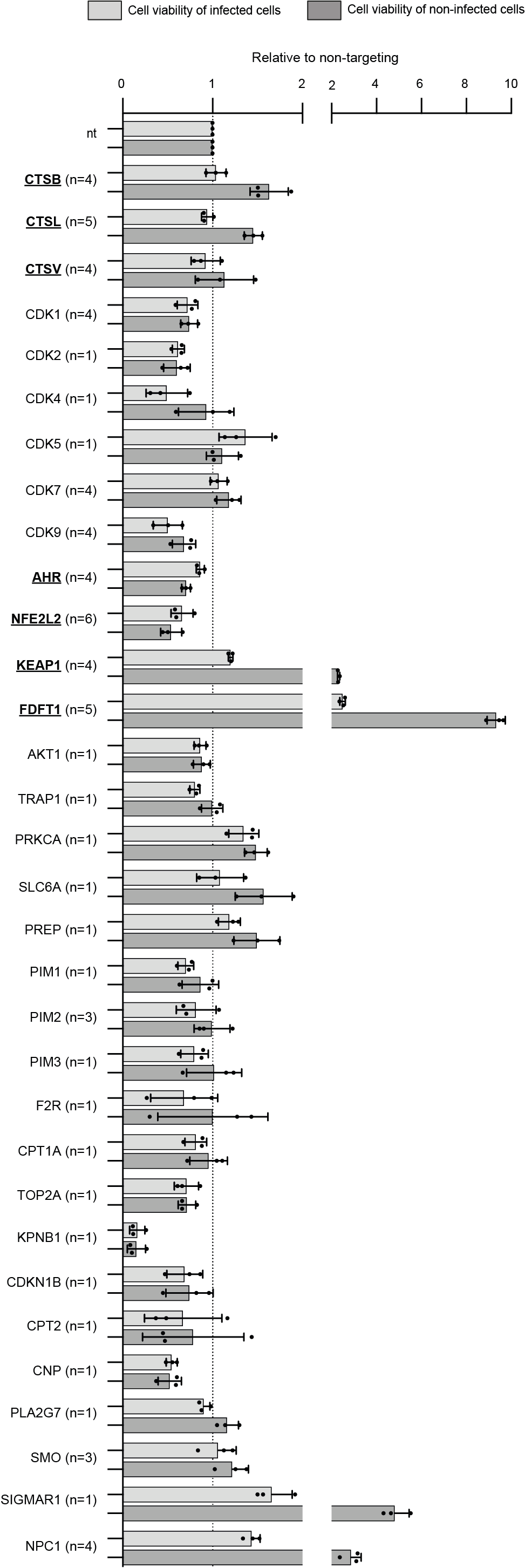


Fig. S4: Cell viability of cells with knock-out of 32 putative host targets of HCoV-229E antivirals. Huh-7.5/Cas9 cells were transduced with sgRNAs targeting 32 putative host targets of HCoV-229E antivirals. The sgRNAs were used for each target. Cells were infected with hCoV-229E for 48 h or left untreated and Firefly luciferase activity was measured. The data was normalized to the respective non‑targeting control (nt). The bars represent the mean of the data acquired with three sgRNA per host factor. Each dot represents the mean of all data acquired using one sgRNA with between one and six biological replicates for each sgRNA (1≤n≤6) as indicated after each host factor name. Each biological replicate was composed of three technical replicates.


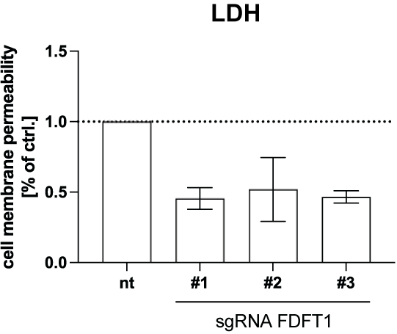


Fig. S5: Determination of cell membrane permeability for Huh‑7.5/F-Luc/Cas9 FDFT1 knock‑out cells. Three Huh‑7.5/F-Luc/Cas9 FDFT1 knock‑out cell lines were generated and infected as described in materials and methods. To quantify cell viability of the knock‑out cells compared to the non‑targeting (nt) control an LDH assay was performed. Absorption at 690 nm was subtracted from absorption detected at 490 nm. Samples were normalized to the nt control. The data was acquired in two independent experiments (n=2) with technical triplicates.


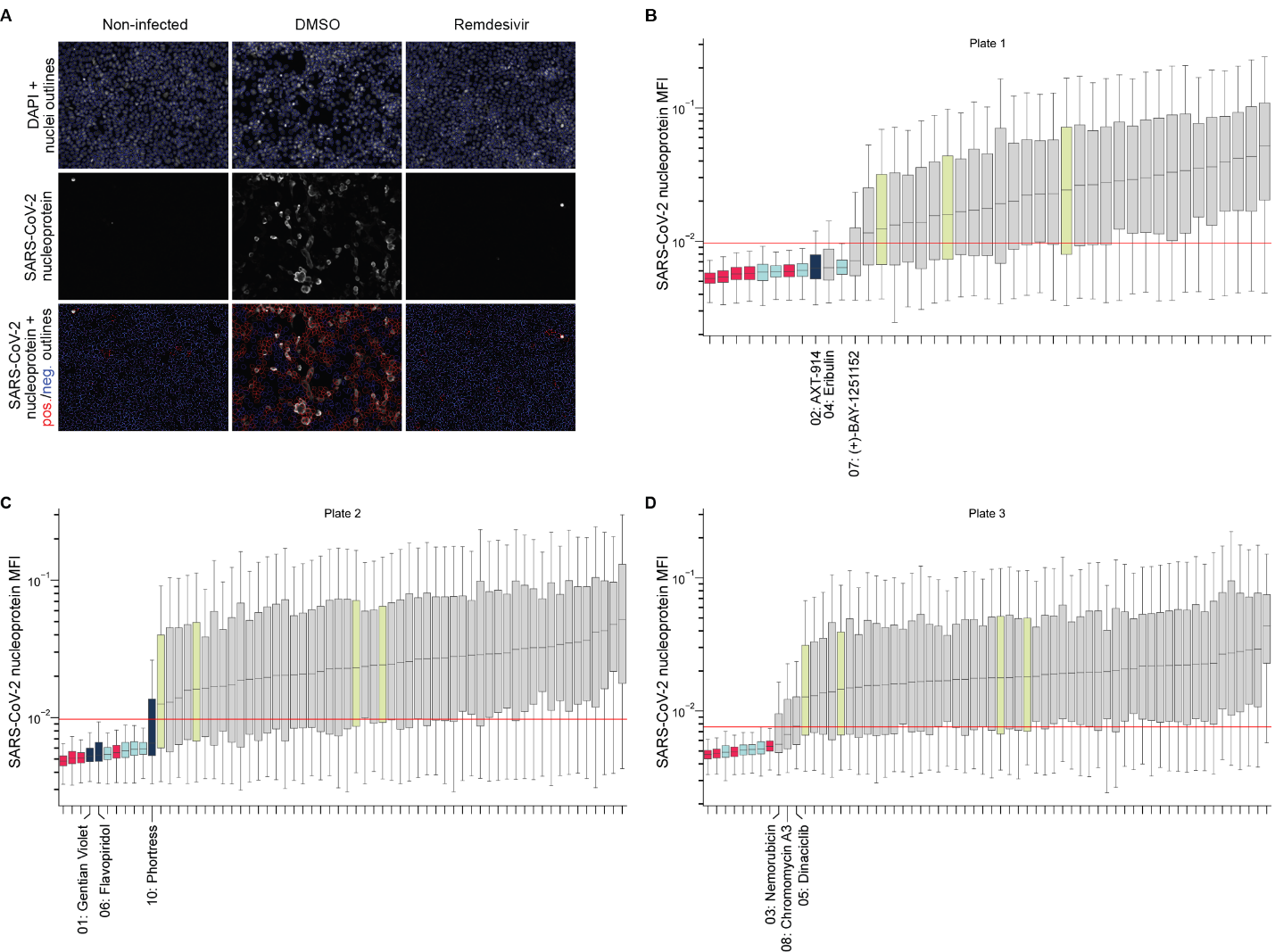


Fig. S6: Imaging workflow for quantification of SARS-CoV-2 infected cells and total cell number. (A) Cell nuclei were segmented based on the DAPI channel, as indicated in the Methods section, and used as substitutes for the cell outlines. The nuclei outlines were overlaid in blue with the DAPI channel (1^st^ row) and used to measure the mean fluorescence intensity (MFI) in the SARS-CoV-2 nucleoprotein channel (2^nd^ row). Cells with a MFI over a set threshold (see red line in panels B, C and D) were counted as infected (red outline, 3^rd^ row); other cells were counted as negative (blue outline, 3^rd^ row). (B-D) Distribution of the cell MFI in the SARS nucleoprotein channel for the individual wells of the three multi-well plates. The threshold (red line) was determined via the 99% quantile of mean fluorescence intensity of the non-infected control wells (b= 0.0096, c = 0.0097, d = 0.0075). The boxplot shows the median MFI with the first and third quartiles. The whisker length corresponds to the last data point inside 1.5 times the IQR (Interquartile range); data outside this range is not displayed. Means of four images per well of one experiment (n=1) with one technical replicate are given. Red = remdesivir, yellow = DMSO, light blue = non-infected/heat-inactivated, dark blue = hit compounds.


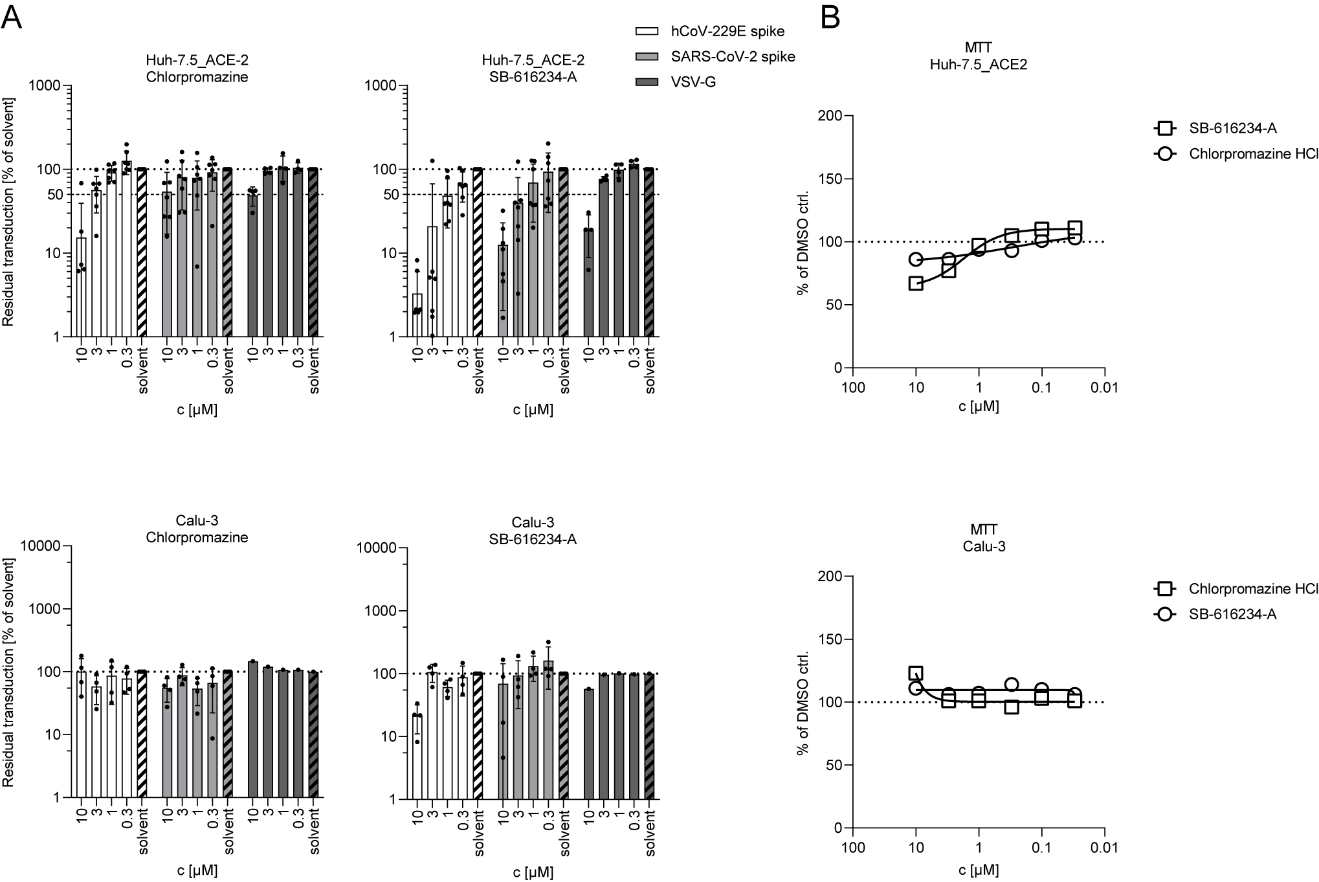


**Fig. S7: Inhibition of HCoV-229E and SARS-CoV-2 glycoprotein-mediated entry.**

**(A)** Huh-7.5/ACE2 cells were pre‑treated with compounds in indicated concentrations or solvent for 2 h and transduced with recombinant VSV-based pseudotypes harboring the glycoproteins of HCoV-229E (white bars), SARS-CoV-2 (grey bars) or VSV (black bars). Firefly luciferase activity was detected after 24 h as a measure of residual virus transduction and RLU were normalized against the solvent control. Cathepsin inhibitor E64 and TMPRSS2-inhibitor Camostat were used as controls. Individual values, means and SD of 5-7 biological replicates with three technical replicates each are depicted (n=2-4 for VSV-G). **(B)** MTT assays were performed to analyze the effect of the respective compounds on cell viability. The data was generated in one experiment (n=1) with technical triplicates.

Dataset S1 (separate file): List of validated compounds inhibiting HCoV‑229E infection of Huh-7.5/F-Luc cells. The 134 hit compounds, named with Reframe identifiers (RFM-ID, column B) are rank ordered (column A) according to their IC_50_ value (column C) determined against HCoV-229E in Huh-7.5 cells. The half maximal cytotoxic dose (CC_50_, column E) as well as the cell viability at the highest dose of 5 µM (column F) are listed. The r-squared values for the curve fits used to calculate the IC_50_ and CC_50_ values are listed in columns D and G, respectively. The SMILES (Simplified molecular-input line-entry system), the name of the drug, any further information provided for the compound and the drug target(s) are listed in columns H to K. Column L lists the targets chosen for consideration for the knockout screening. Column M lists the corresponding RPKM (reads per million mapped reads) values determined in Huh-7.5 cells. Information on the mode of action, clinical phase, and screening status according to reframed.org (December 2020). Columns Q and R list the results of the anti-SARS-CoV-2 screening. All numerical data was acquired in this study and additional descriptive information as well as host-targets were retrieved from reframedb.org, drugbank.com, pubchem.ncbi.nlm.nih.gov, guidetopharmacology.org, medchemexpress.com and pubmed.ncbi.nlm.nih.gov.

Primer sequences for the generation of sgRNAs to induce CRISPR/Cas9‑mediated knock‑out of host targets

| Name | sgRNA number | Forward  5’ 🡪 3’ | Reverse  5’ 🡪 3’ |
| --- | --- | --- | --- |
| Non-targeting control (target: HCV E2) |  | CACCGTGGTGGTGGGAACGACCGA | AAACTCGGTCGTTCCCACCACCAC |
| AHR | 1 | CACCGAAGTCGGTCTCTATGCCGCT | AAACAGCGGCATAGAGACCGACTTC |
| AHR | 2 | CACCGGTCCAACTCTGTATTAAGT | AAACACTTAATACAGAGTTGGACC |
| AHR | 3 | CACCGTCAAGTCAAATCCTTCCAAG | AAACCTTGGAAGGATTTGACTTGAC |
| KPNB1 | 1 | CACCGCACATGAAGGAGTCGACAT | AAACATGTCGACTCCTTCATGTGC |
| KPNB1 | 2 | CACCGATCTTGGCAAATATAACCGA | AAACTCGGTTATATTTGCCAAGATC |
| KPNB1 | 3 | CACCGCATTGGGTACAGAAACTTAC | AAACGTAAGTTTCTGTACCCAATGC |
| CDK1 | 1 | CACCGTACTTGGATTCTATCCCTCC | AAACGGAGGGATAGAATCCAAGTAC |
| CDK1 | 2 | CACCGACAAAACACAATCCCCTGT | AAACACAGGGGATTGTGTTTTGTC |
| CDK1 | 3 | CACCGTCATGTGTATATACTCTGAT | AAACATCAGAGTATATACACATGAC |
| CDK2 | 1 | CACCGCAGAAACAAGTTGACGGGAG | AAACCTCCCGTCAACTTGTTTCTGC |
| CDK2 | 2 | CACCGGCGCTTAAGAAAATCCGCC | AAACGGCGGATTTTCTTAAGCGCC |
| CDK2 | 3 | CACCGCTCTCCCGTCAACTTGTTTC | AAACGAAACAAGTTGACGGGAGAGC |
| CDK4 | 1 | CACCGGTGGCTTTACTGAGGCGAC | AAACGTCGCCTCAGTAAAGCCACC |
| CDK4 | 2 | CACCGAGCCACTGGCTCATATCGAG | AAACCTCGATATGAGCCAGTGGCTC |
| CDK4 | 3 | CACCGAAATTGGTGTCGGTGCCTA | AAACTAGGCACCGACACCAATTTC |
| CDK5 | 1 | CACCGGCCTTGAACACAGTTCCGT | AAACACGGAACTGTGTTCAAGGCC |
| CDK5 | 2 | CACCGTAGCCGCAATGTGCTACACA | AAACTGTGTAGCACATTGCGGCTAC |
| CDK5 | 3 | CACCGTCAGCTGAGTAACAGCGGAC | AAACGTCCGCTGTTACTCAGCTGAC |
| CDK7 | 1 | CACCGTCGGGCTTTACGGCGCCGGA | AAACTCCGGCGCCGTAAAGCCCGAC |
| CDK7 | 2 | CACCGACTTCACGTCCAGAGCCATC | AAACGATGGCTCTGGACGTGAAGTC |
| CDK7 | 3 | CACCGGAGTCGGGCTTTACGGCGC | AAACGCGCCGTAAAGCCCGACTCC |
| CDK9 | 1 | CACCGTACGAGAAGCTCGCCAAGAT | AAACATCTTGGCGAGCTTCTCGTAC |
| CDK9 | 2 | CACCGAAGGTGCCTTGGCCGATCT | AAACAGATCGGCCAAGGCACCTTC |
| CDK9 | 3 | CACCGCTGGCCGGTCTTGCGGTGCC | AAACGGCACCGCAAGACCGGCCAGC |
| CPT1A | 1 | CACCGCAGTTCACGGTCACTCCGGA | AAACTCCGGAGTGACCGTGAACTGC |
| CPT1A | 2 | CACCGCTCCGGACGGGATTGACCTG | AAACCAGGTCAATCCCGTCCGGAGC |
| CPT1A | 3 | CACCGAGCCGCAGGTCAATCCCGTC | AAACGACGGGATTGACCTGCGGCTC |
| CPT2 | 1 | CACCGGGGCTCCCGGACCAACCGC | AAACGCGGTTGGTCCGGGAGCCCC |
| CPT2 | 2 | CACCGCGTGCCCACCATGCACTACC | AAACGGTAGTGCATGGTGGGCACGC |
| CPT2 | 3 | CACCGTGGGCACGATGCTGCGCTGC | AAACGCAGCGCAGCATCGTGCCCAC |
| PRKCA | 1 | CACCGCTCCACGGCGTCTCAGGACG | AAACCGTCCTGAGACGCCGTGGAGC |
| PRKCA | 2 | CACCGCAACCGCTTCGCCCGCAAAG | AAACCTTTGCGGGCGAAGCGGTTGC |
| PRKCA | 3 | CACCGTTCTTGTCCGGGTGCGGATA | AAACTATCCGCACCCGGACAAGAAC |
| CTSB | 1 | CACCGTCAACAAACGGAATACCACG | AAACCGTGGTATTCCGTTTGTTGAC |
| CTSB | 2 | CACCGTGTTGGCCAATGCCCGGAGC | AAACGCTCCGGGCATTGGCCAACAC |
| CTSB | 3 | CACCGTTGACCAGCTCATCCGACA | AAACTGTCGGATGAGCTGGTCAAC |
| CTSL | 1 | CACCGTTATGAGGCCCCCAGATCTG | AAACCAGATCTGGGGGCCTCATAAC |
| CTSL | 2 | CACCGCTTTCAAAACCGTAAGCCC | AAACGGGCTTACGGTTTTGAAAGC |
| CTSL | 3 | CACCGCCAGGAACCTCTGTTTTATG | AAACCATAAAACAGAGGTTCCTGGC |
| CTSV | 1 | CACCGTCAAATTTTGGAACAGCGG | AAACCCGCTGTTCCAAAATTTGAC |
| CTSV | 2 | CACCGCATGAATCTTTCGCTCGTCC | AAACGGACGAGCGAAAGATTCATGC |
| CTSV | 3 | CACCGTGGATACAAAGTGGTACCAG | AAACCTGGTACCACTTTGTATCCAC |
| SLC6A4 | 1 | CACCGCGTGTGTCATCTCCCGCACC | AAACGGTGCGGGAGATGACACACGC |
| SLC6A4 | 2 | CACCGAGTCCGGGCAAATATCCAAT | AAACATTGGATATTTGCCCGGACTC |
| SLC6A4 | 3 | CACCGCAGCTATCAGCGTGTGAAGA | AAACTCTTCACACGCTGATAGCTGC |
| CNP | 1 | CACCGAAGGCTCCTCGAGCGCCGG | AAACCCGGCGCTCGAGGAGCCTTC |
| CNP | 2 | CACCGGAGTACAAGCGGCTCGATG | AAACCATCGAGCCGCTTGTACTCC |
| CNP | 3 | CACCGCGGCAAGTCCACGCTGGCAC | AAACGTGCCAGCGTGGACTTGCCGC |
| TRAP1 | 1 | CACCGTGCTGCTGATAATCGAGTGC | AAACGCACTCGATTATCAGCAGCAC |
| TRAP1 | 2 | CACCGTTCAGCACGCAGACCGCCG | AAACCGGCGGTCTGCGTGCTGAAC |
| TRAP1 | 3 | CACCGTCGTCCTGCCTGCAAGCTCC | AAACGGAGCTTGCAGGCAGGACGAC |
| PLA2G7 | 1 | CACCGGCCAAACTAAAATCCCCCG | AAACCGGGGGATTTTAGTTTGGCC |
| PLA2G7 | 2 | CACCGAATAAGGCCCATTTCCCCG | AAACCGGGGAAATGGGCCTTATTC |
| PLA2G7 | 3 | CACCGTCAAGGCGATCATTATCTT | AAACAAGATAATGATCGCCTTGAC |
| PREP | 1 | CACCGAAATTTGTGACCCTTACGCC | AAACGGCGTAAGGGTCACAAATTTC |
| PREP | 2 | CACCGCCATCGTCAGACAGTATGT | AAACACATACTGTCTGACGATGGC |
| PREP | 3 | CACCGCCTGTACATATAATACTCGC | AAACGCGAGTATTATATGTACAGGC |
| CDKN1B | 1 | CACCGATCCGTCGGACAGCCAGACG | AAACCGTCTGGCTGTCCGACGGATC |
| CDKN1B | 2 | CACCGCACTCGTACTTGCCCTCTAG | AAACCTAGAGGGCAAGTACGAGTGC |
| CDKN1B | 3 | CACCGAGTTCTACTACAGACCCCCG | AAACCGGGGGTCTGTAGTAGAACTC |
| PIM1 | 1 | CACCGTGGCGTGCAGGTCGTTGCA | AAACTGCAACGACCTGCACGCCAC |
| PIM1 | 2 | CACCGTACCAGGTGGGCCCGCTAC | AAACGTAGCGGGCCCACCTGGTAC |
| PIM1 | 3 | CACCGAAGCCGCCGCTGCCCAGTAG | AAACCTACTGGGCAGCGGCGGCTTC |
| PIM2 | 1 | CACCGTGTTGACCAAGCCTCTACAG | AAACCTGTAGAGGCTTGGTCAACAC |
| PIM2 | 2 | CACCGAGGGGGCCGAGTCGATACT | AAACAGTATCGACTCGGCCCCCTC |
| PIM2 | 3 | CACCGCGCACCTCTGCGGGCCCGG | AAACCCGGGCCCGCAGAGGTGCGC |
| PIM3 | 1 | CACCGCGGGAGGTGGTCCACGCCGC | AAACGCGGCGTGGACCACCTCCCGC |
| PIM3 | 2 | CACCGCGCACCTCTGCGGGCCCGG | AAACCCGGGCCCGCAGAGGTGCGC |
| PIM3 | 3 | CACCGCGGGTAGCCGCATCGCCGA | AAACTCGGCGATGCGGCTACCCGC |
| SMO | 1 | CACCGTAGCGCAGCGGCTCGCAGG | AAACCCTGCGAGCCGCTGCGCTAC |
| SMO | 2 | CACCGTTGTAGCGCAGCGGCTCGC | AAACGCGAGCCGCTGCGCTACAAC |
| SMO | 3 | CACCGCCTCGAGCGGGAACGCGACC | AAACGGTCGCGTTCCCGCTCGAGGC |
| FDFT1 | 1 | CACCGACCTGGTGCGCTTCCGGATC | AAACGATCCGGAAGCGCACCAGGTC |
| FDFT1 | 2 | CACCGCCAGGTTGTAGAACTCTTCG | AAACCGAAGAGTTCTACAACCTGGC |
| FDFT1 | 3 | CACCGCTGTTATCCAGGCGCTGGA | AAACTCCAGCGCCTGGATAACAGC |
| SIGMAR1 | 1 | CACCGACTGCGCTATCTCTTCGCGC | AAACGCGCGAAGAGATAGCGCAGTC |
| SIGMAR1 | 2 | CACCGCGGTGCTGACCCAGGTCGTC | AAACGACGACCTGGGTCAGCACCGC |
| SIGMAR1 | 3 | CACCGTCAGCACCGCTGCGACAGCC | AAACGGCTGTCGCAGCGGTGCTGAC |
| F2R | 1 | CACCGCGGGCCGCACAGACTGAAGC | AAACGCTTCAGTCTGTGCGGCCCGC |
| F2R | 2 | CACCGCGGGTGCGGGCAGACAACAG | AAACCTGTTGTCTGCCCGCACCCGC |
| F2R | 3 | CACCGGAGCTGGTCAAATATCCGG | AAACCCGGATATTTGACCAGCTCC |
| TOP2A | 1 | CACCGCTCCGCCCAGACACCTACAT | AAACATGTAGGTGTCTGGGCGGAGC |
| TOP2A | 2 | CACCGATGCTGCGGACAACAAACAA | AAACTTGTTTGTTGTCCGCAGCATC |
| TOP2A | 3 | CACCGCATCATAGTTACTAGAAGTT | AAACAACTTCTAGTAACTATGATGC |
| AKT1 | 1 | CACCGCGACGTGGCTATTGTGAAGG | AAACCCTTCACAATAGCCACGTCGC |
| AKT1 | 2 | CACCGTGGCTACAAGGAGCGGCCGC | AAACGCGGCCGCTCCTTGTAGCCAC |
| AKT1 | 3 | CACCGTGTTGAGGGGAGCCTCACGT | AAACACGTGAGGCTCCCCTCAACAC |
| NFE2L2 | 1 | CACCGTAGTTGTAACTGAGCGAAAA | AAACTTTTCGCTCAGTTACAACTAC |
| NFE2L2 | 2 | CACCGGAGTAGTTGGCAGATCCAC | AAACGTGGATCTGCCAACTACTCC |
| NFE2L2 | 3 | CACCGTGATTTAGACGGTATGCAAC | AAACGTTGCATACCGTCTAAATCAC |
| KEAP1 | 1 | CACCGTACGCCTCCACTGAGTGCA | AAACTGCACTCAGTGGAGGCGTAC |
| KEAP1 | 2 | CACCGACTGGGCGGCCGGTGCATCC | AAACGGATGCACCGGCCGCCCAGTC |
| KEAP1 | 3 | CACCGCCTGGAGGATCATACCAAGC | AAACGCTTGGTATGATCCTCCAGGC |
| NPC1 | 1 | CACCGTCAAAAACTGACTCTGTCGA | AAACTCGACAGAGTCAGTTTTTGAC |
| NPC1 | 2 | CACCGTCAAAAACTGACTCTGTCGA | AAACTCGACAGAGTCAGTTTTTGAC |
| NPC1 | 3 | CACCGCAGGGCCTTGTCATTACTTG | AAACCAAGTAATGACAAGGCCCTGC |

**Entry assay with VSV-based pseudotypes**

Production of recombinant VSV-based pseudotypes was performed based on a previously described protocol (1). In brief, for the reproduction of the initial rVSVΔG GFP G stock, BHK-G43 cells were incubated with 10^-9^ M mifepristone for 6 h (37 °C, 5 % CO_2_) to induce G glycoprotein expression. VSVΔG G was added 1:100 to the cells for 24 h, before the supernatant was harvested, cleared from residual cells by centrifugation at 1,200 x g at 4 °C for 15 min, aliquoted and stored at -80 °C. The stocks were titrated on HEK 293T cells.

For production of pseudotyped VSV particles, 4.8x10^6^ HEK 293T cells were seeded in a 10 cm dish and transfected with 6 µg/µL of env plasmid using Lipofectamine 2000 (ratio 1:1). After 4-6 h at 37 °C and 5 % CO_2_ medium was exchanged. The next day, the HEK 293T cells were transduced with rVSVΔG GFP G at an MOI of 3 and incubated at 37 °C with 5 % CO_2_. After 2 h the cells were washed with PBS and an anti VSV G antibody (I1, produced from CRL 2700 mouse hybridoma cells) was added to the cells for neutralization. Cells were incubated overnight; the supernatants were collected and cellular debris removed by centrifugation (2,000 x g / 10 min). The pseudoparticles were aliquoted and stored at -80 °C. The stocks were titrated on the target cells.

For infection assays, Huh-7.5/ACE2 (2x10^4^ cells/well; seeded 24 h before treatment) and Calu-3 (4.5x10^4^ cells/well; seeded 48 h before treatment) cells were seeded in 96 well plates. The next day, cells were pre-treated with the respective compounds or solvent for 2 h and then inoculated with recombinant VSV based pseudotypes harboring the glycoproteins of HCoV-229E, SARS-CoV-2 or VSV for 3-4 h. Firefly luciferase activity was detected after 16-18 h as a measure of residual virus transduction and RLU were normalized against the solvent control.

**MTT assay for cell viability quantification**

To quantify the cytotoxicity of selected compounds in the VSV pseudotype assay, MTT assays were performed. Cells were incubated with 10 µM puromycin for 24 hours as a positive control for cytotoxicity. Compound-treated cells were incubated with medium containing 1 mg/ml MTT (Sigma‑Aldrich #M5655) for 30 minutes at 37 °C. Absorbance at 570 and 630 nm was measured with a BioTek Synergy 2 plate reader (Biotek Instruments).

**RNA sequencing**

Total RNA from plated Huh-7.5 cells was extracted using a NucleoSpin RNA kit (Macherey Nagel) according to the manufacturer’s instructions, and mRNA integrity checking was performed using an Agilent Bioanalyzer. RNA was used to generate a sequencing library using a ScriptSeqv2 kit (Illumina) according to the manufacturer’s protocol and library sequencing was performed using the Illumina HiSeq 2500 instrument. The generated FASTQ file was then mapped against the hg19 human reference genomes, with annotated gene and mRNA tracks, using CLC Genomics Workbench (QIAGEN). Count data mapped to individual human gene loci was used to calculate normalized transcript expression levels for all expressed genes (reads per kilobase, per million mapped bases: RPKM).

**Synthesis of P‑3622**

**General Experimental Information**

Commercial reagents were used as received, and all other reagents were prepared using known literature procedures. All solvents used for reactions, workups, and purifications had the HPLC purity grade. Dried solvents were purchased in water free form (99.5%, extra dry, absolute, AcroSeal^TM^, ACROS Organics^TM^) and used as received.

Reactions were either monitored by Liquid Chromatography-coupled Mass Spectrometry (LCMS) analysis or thin-layer chromatography (TLC) on “TLC Silica gel 60 F254” plates (Merck) and visualized by staining with aqueous basic KMnO_4_. LCMS was conducted with an Agilent® 1260 HPLC System with a DAD detector and an Agilent® 6130 quadrupole mass detector with Electro Spray Ionization (ESI) (MeCN/H_2_O + 0.1% formic acid). Flash chromatography was performed on silica gel 60 (technical grade, pore size 60 Å, 40–63 µm, 230–400 mesh, Supelco®).

For Nuclear Magnetic Resonance (NMR) spectroscopic analysis Bruker Avance III or Bruker Avance III HD spectrometers were employed and ^1^H NMR spectra were recorded at 500 MHz, 600 MHz or 700 MHz, respectively 126 MHz, 151 MHz and 176 MHz for ^13^C NMR spectra. Chemical shifts are reported in parts per million (ppm) using the residual non-deuterated solvent resonance for proton measurements and the solvent resonance for carbon measurements as an internal standard (CDCl_3_: ^1^H = 7.26 ppm and ^13^C = 77.2 ppm). Data are reported as follows: chemical shift (multiplicity (s = singlet, t = triplet, q = quartet, m = multiplet), coupling constant(s) in Hz, integration).

**Preparation of P-3622**

Compound P-3622 was not commercially available and was therefore re-synthesized by us. We conducted an aldol condensation of *p*-methoxyphenyl-acetonitrile **1** with propiophenone **2** to furnish a separable mixture of the acrylonitriles ***E*/*Z*-3** (figure 1).

Synthesis of P-3622; b) NOE correlations for determination of the double bond geometry of the anisole isomers (4).

Demethylation of the major *trans*-stilben isomer in refluxing pyridinium chloride yielded an isomeric mixture of the phenols ***E*/*Z*-4**. Following their separation, the double bond geometry of the isomers was ensured by NOE experiments. The *trans*-stilben ***Z*-4** was then alkylated to provide P-3622 (***Z*-5**) for further investigations.

**Preparation of 3-(4-chlorophenyl)-2-(4-methoxyphenyl)pent-2-enenitrile (3)**

A suspension of NaH (60% in mineral oil; 816 mg, 20.4 mmol, 1.02 eq) in xylene (7 ml) was heated to reflux under an inert gas atmosphere and carefully treated with a solution of 2-(4-methoxy­phenyl)­aceto­nitrile (**1**; 2.941 g, 20.0 mmol, 1.00 eq) in xylene (5 ml) in a manner to keep the delayed hydrogen evolution under control. Afterwards a solution of 1-(4-chlorophenyl)propan-1-one (**2**; 4.047 g, 24.0 mmol, 1.20 eq) in xylene (6 ml) was added dropwise, the mixture was refluxed for further 30 minutes, cooled to ambient temperature and distributed between MTBE (100 ml) and a half saturated aqueous solution of NH_4_Cl (75 ml). The aqueous phase was extracted one further time with MTBE (100 ml), the combined organic extracts were dried over MgSO_4_ and all volatiles were removed under reduced pressure. The deep brown crude reaction mixture was filtered over SiO_2_ and eluted gradually with EtOAc/hexanes (0% -> 20%). The combined fractions, which contained the geometrical isomers of the desired product, were recrystallized from Et_2_O/*n*-hexane to provide the title compound (1.326 g, 4.453 mmol) as a mixture of isomers in form of colorless crystals. Flash chromatography of the resulting mother liquor separated the diastereomers of the title compound (581 mg, 1.95 mmol of main isomer ***Z*-3**; respectively 655 mg, 2.20 mmol of ***E*-3**; in total 43%) as an off-white solid.

**Major isomer** **(*Z*-3)**

**^1^H NMR (700 MHz, CDCl_3_, 298 K):** *δ* = 7.43 (m_c_, 2 H), 7.38 (m_c_, 2 H), 7.33 (m_c_, 2 H), 6.96 (m_c_, 2 H), 3.85 (s, 3 H), 2.58 (q, *J* = 7.5 Hz, 2 H), 0.93 (t, *J* = 7.5 Hz, 3 H) ppm.

**^13^C NMR (176 MHz, CDCl_3_, 298 K):** *δ* = 160.10, 160.06, 137.7, 136.2, 130.3, 129.4, 129.1, 126.6, 119.4, 114.4, 112.3, 55.6, 27.7, 13.0 ppm.

**Minor isomer (*E*-3)**

**^1^H NMR (700 MHz, CDCl_3_, 298 K):** *δ* = 7.23 (m_c_, 2 H), 7.00 (m_c_, 4 H), 6.70 (m_c_, 2 H), 3.75 (s, 3 H), 2.92 (q, *J* = 7.6 Hz, 2 H), 1.06 (t, *J* = 7.6 Hz, 3 H) ppm.

**^13^C NMR (176 MHz, CDCl_3_, 298 K):** *δ* = 159.5, 158.3, 136.6, 134.4, 130.1, 129.0, 125.9, 118.9, 114.0, 111.6, 55.4, 32.2, 12.8 ppm.

**ESI-HRMS:** calc. for C_18_H_17_ClNO^+^ [M + H]^+^: *m*/*z* = 298.0993; found *m*/*z* = 298.0993.

**Preparation of 3-(4-chlorophenyl)-2-(4-hydroxyphenyl)pent-2-enenitrile (4)**

The major isomer of anisol **3** (563 mg, 1.89 mmol, 1.00 eq) was refluxed in pyridinium chloride (4.00 g, 34.6 mmol, 18.31 eq) for 6 h or till complete disappearance of the starting material. The cold reaction mixture was diluted with MTBE (100 ml), a saturated aqueous solution of K_2_CO_3_ (75 ml) was added carefully and the phases were separated. The aqueous phase was further extracted with PhMe (2 x 75 ml), the combined organic extracts were dried over Na_2_SO_4_, and concentrated under reduced pressure. The crude mixture contained both geometrical isomers of the phenol **4**, which were separated by flash chromatography 10-40% EtOAc/hexanes) to yield the desired isomers (357 mg, 1.258 mmol; respectively 78 mg, 274.8 µmol; in total 81%) as colorless solids.

**Major isomer *Z*-4**

**^1^H NMR (500 MHz, CDCl_3_, 298 K):** *δ* = 7.43 (m_c_, 2 H), 7.38 (m_c_, 2 H), 7.28 (m_c_, 2 H), 6.89 (m_c_, 2 H), 5.31 (brs, 1 H), 2.58 (q, *J* = 7.5 Hz, 2 H), 0.93 (t, *J* = 7.5 Hz, 3 H) ppm.

**^13^C NMR (125 MHz, CDCl_3_, 298 K):** *δ* = 160.10, 156.4, 137.6, 136.2, 135.7, 129.9, 129.6, 127.2, 116.2, 112.4, 112.3, 28.1, 13.3 ppm

**Minor isomer *E*-4**

**^1^H NMR (500 MHz, CDCl_3_, 298 K):** *δ* = 7.23 (m_c_, 2 H), 6.99 (m_c_, 2 H), 6.95 (m_c_, 2 H), 6.64 (m_c_, 2 H), 5.18 (brs, 1 H), 2.91 (q, *J* = 7.6 Hz, 2 H), 1.06 (t, *J* = 7.6 Hz, 3 H) ppm.

**^13^C NMR (125 MHz, CDCl_3_, 298 K):** *δ* = 158.7, 155.7, 136.5, 134.5, 131.1, 130.1, 129.0, 126.0, 118.9, 115.6, 111.4, 32.2, 12.8 ppm.

**ESI-HRMS:** calc. for C_17_H_15_ClNO^+^ [M + H]^+^: *m*/*z* = 284.0837; found *m*/*z* = 284.0837.

**Preparation of *Z*-3-(4-chlorophenyl)-2-(4-(2-(diethylamino)ethoxy)phenyl)pent-2-enenitrile (P-3622; *Z*-5)**

A solution of the major phenol isomer ***Z*-4** (317 mg, 1.258 mmol, 1.00 eq) in dry acetone (5 ml) was treated with finely divided K_2_CO_3_ (869 mg, 6.290 mmol, 5.00 eq) under an inert gas atmosphere followed by addition of 2-bromo-*N*,*N*-diethylethan-1-aminium chloride (341 mg, 1.573 mmol, 1.25 eq) and the slurry was vigorously stirred for 14 h at 50 °C. After removal of all volatiles, the mixture was distributed between MTBE (50 ml) and diluted brine (30 ml total; 1:1 mixture of brine and deionized water) with the same volume of water). The aqueous phase was extracted one further time with MTBE (50 ml), the combined organic extracts were dried over MgSO_4_ and all volatiles were removed under reduced pressure. The crude product was purified by flash chromatography (1−10% Et_3_N/PhMe) to provide P-3622 (347 mg, 987.7 µmol, 72%) as a colorless solid.

**P-3622 (*Z*-5)**

**^1^H NMR (500 MHz, CDCl_3_, 298 K):** *δ* = 7.42 (m_c_, 2 H), 7.37 (m_c_, 2 H), 7.31 (m_c_, 2 H), 6.95 (m_c_, 2 H), 4.08 (t, *J* = 6.3 Hz, 2 H), 2.90 (t, *J* = 6.3 Hz, 2 H), 2.65 (q, *J* = 7.2 Hz, 4 H), 2.57 (q, *J* = 7.5 Hz, 2 H), 1.08 (q, *J* = 7.2 Hz, 6 H), 0.92 (t, *J* = 7.5 Hz, 3 H) ppm.

**^13^C NMR (126 MHz, CDCl_3_, 298 K):** *δ* = 160.1, 159.3, 137.7, 135.2, 130.3, 129.4, 129.1, 126.5, 119.4, 115.0, 112.3, 66.8, 51.8, 48.0, 27.7, 12.9, 11.9 ppm.

**ESI-HRMS:** calc. for C_23_H_28_ClN_2_O^+^ [M + H]^+^: *m*/*z* = 383.1885; found *m*/*z* = 383.1887.

**NMR spectra**


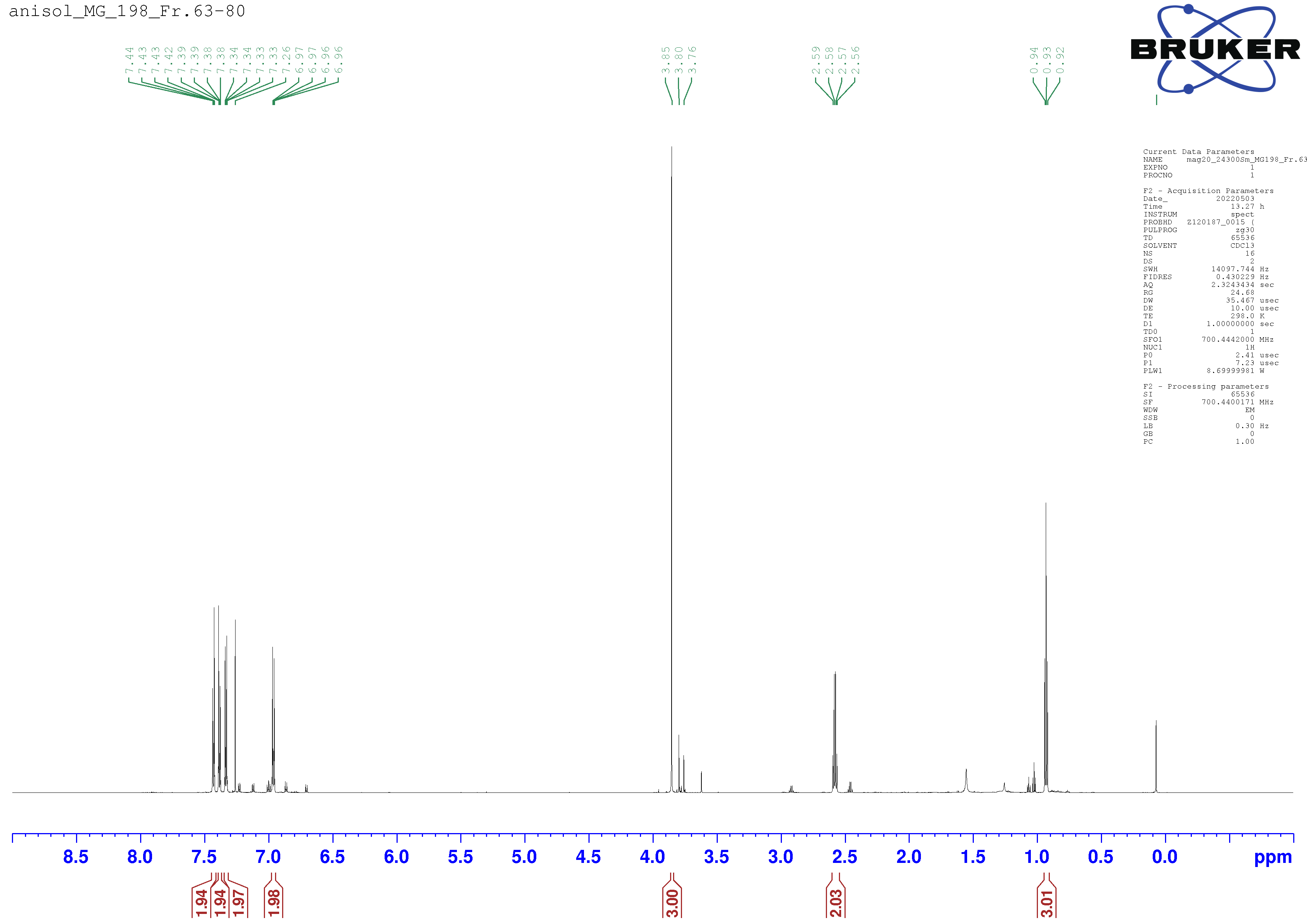


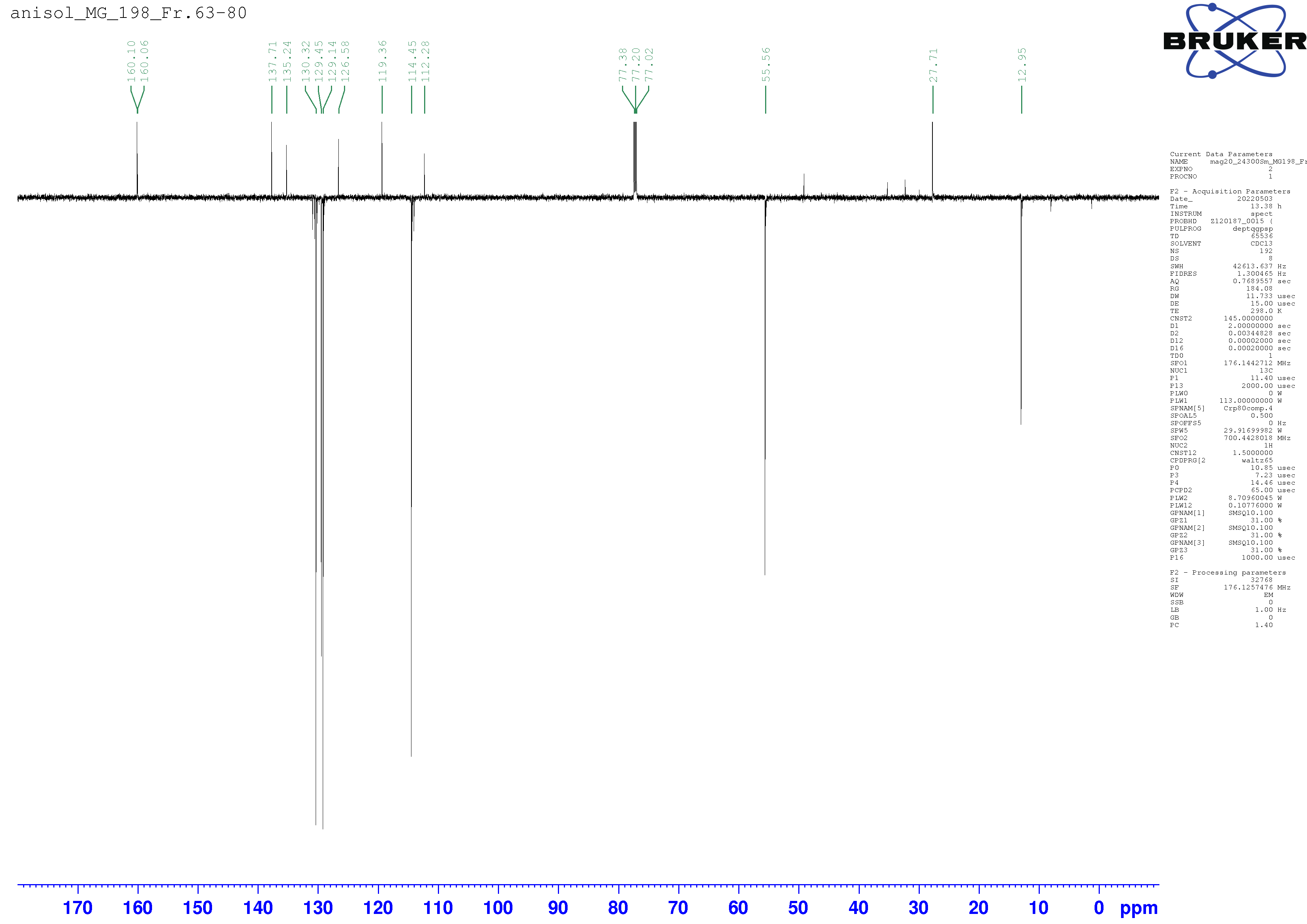


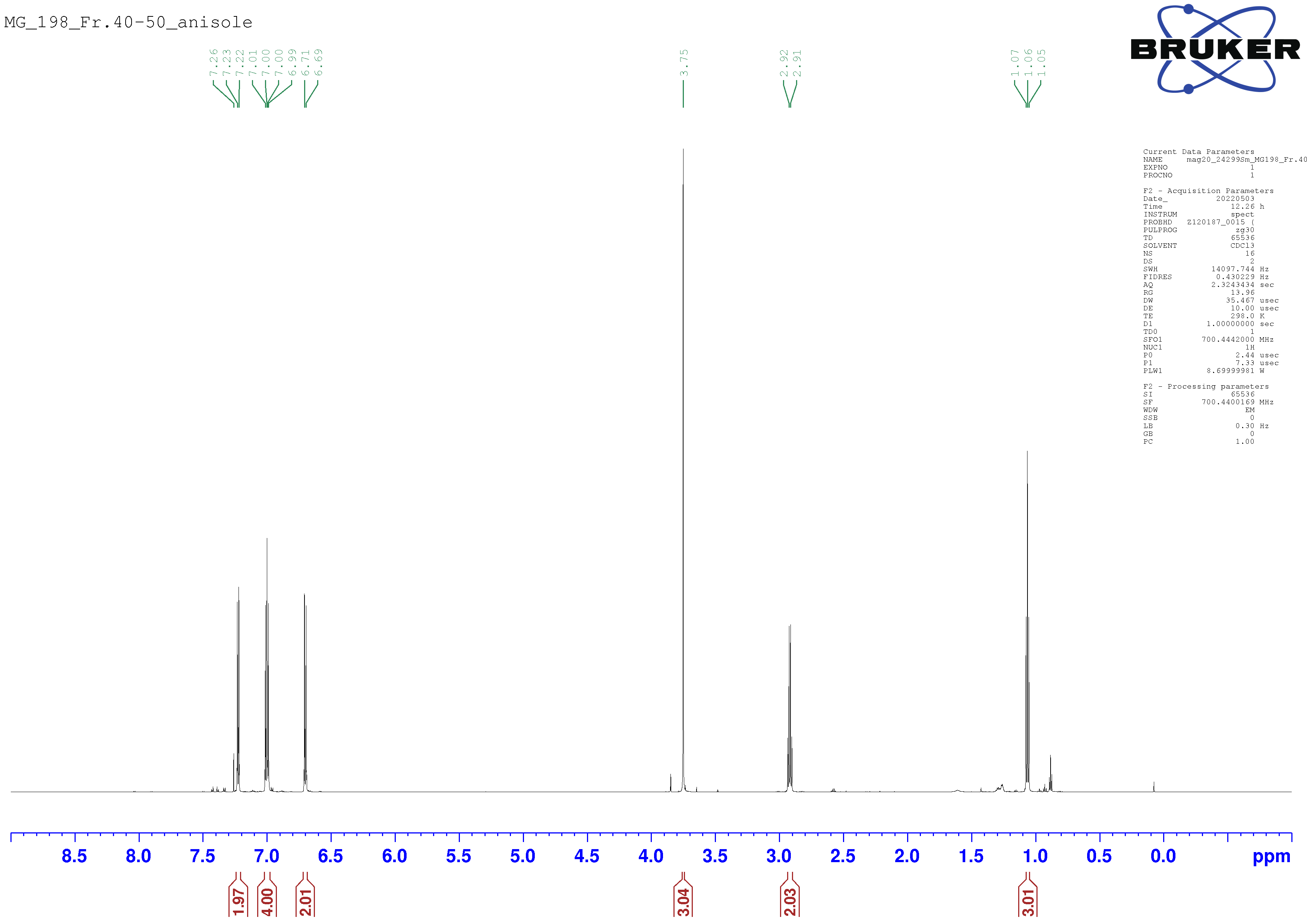


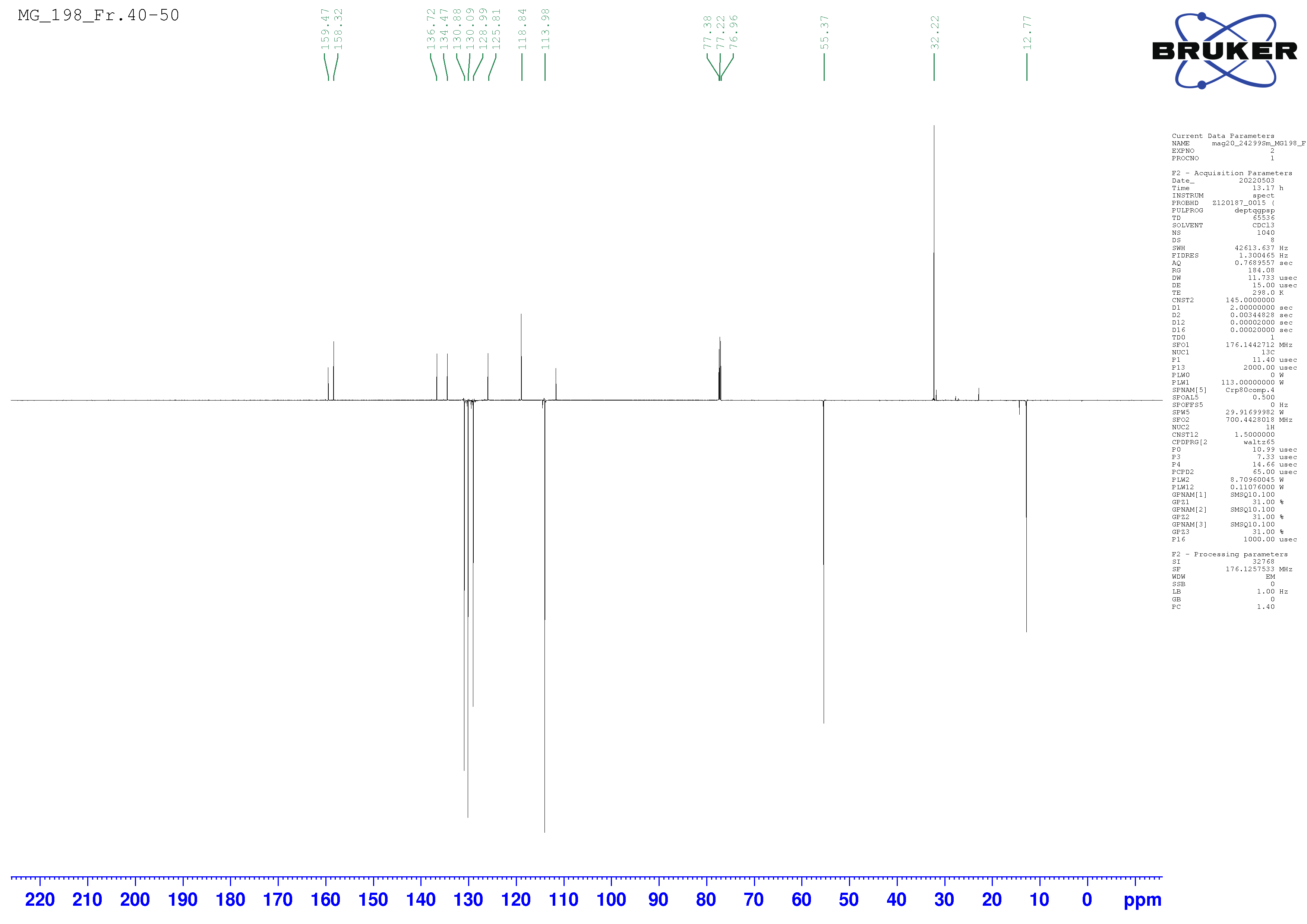


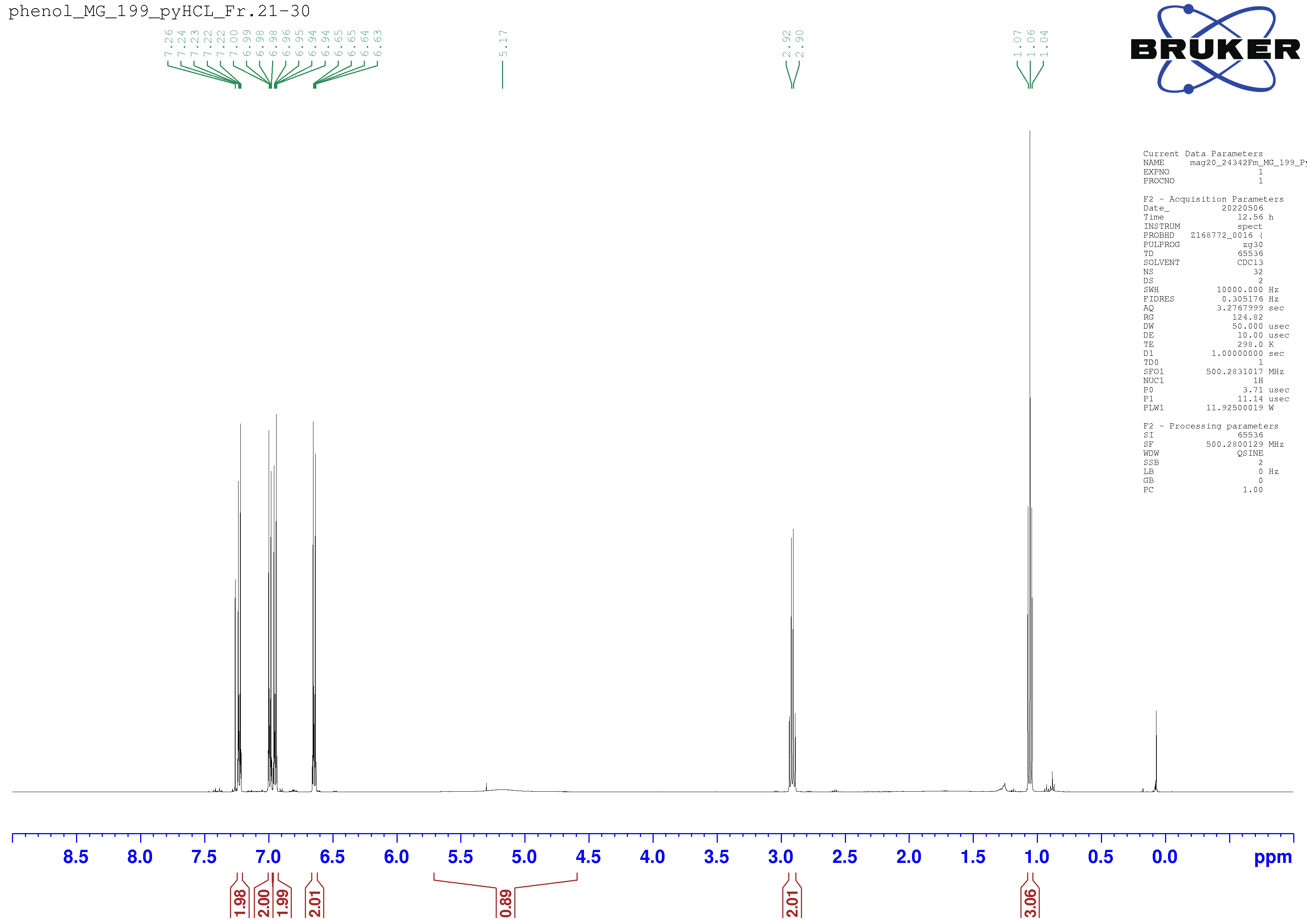


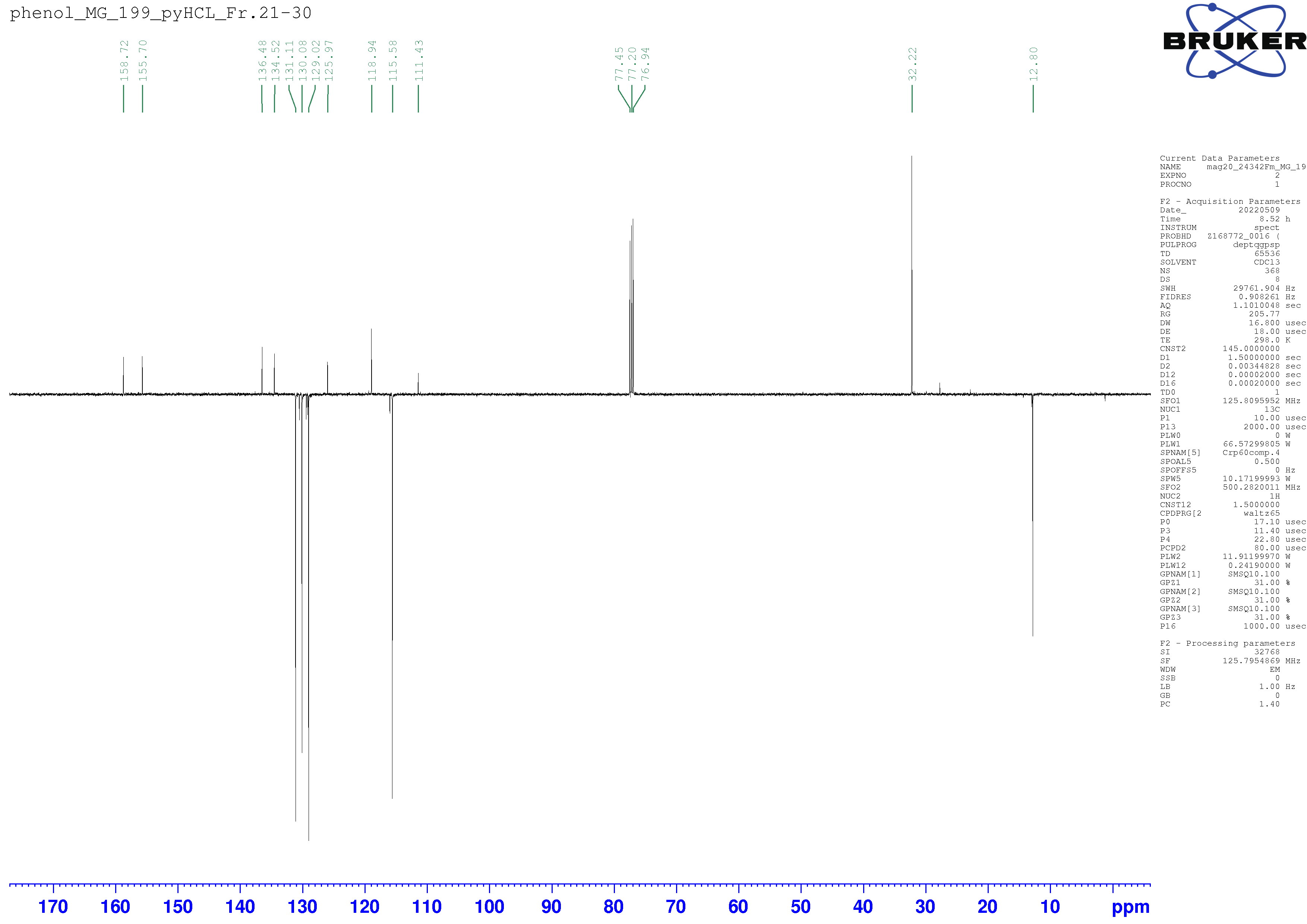


**2D spectra for assignment and NOE correlations of *E*/*Z*-4 as isomeric mixture**

**COSY**


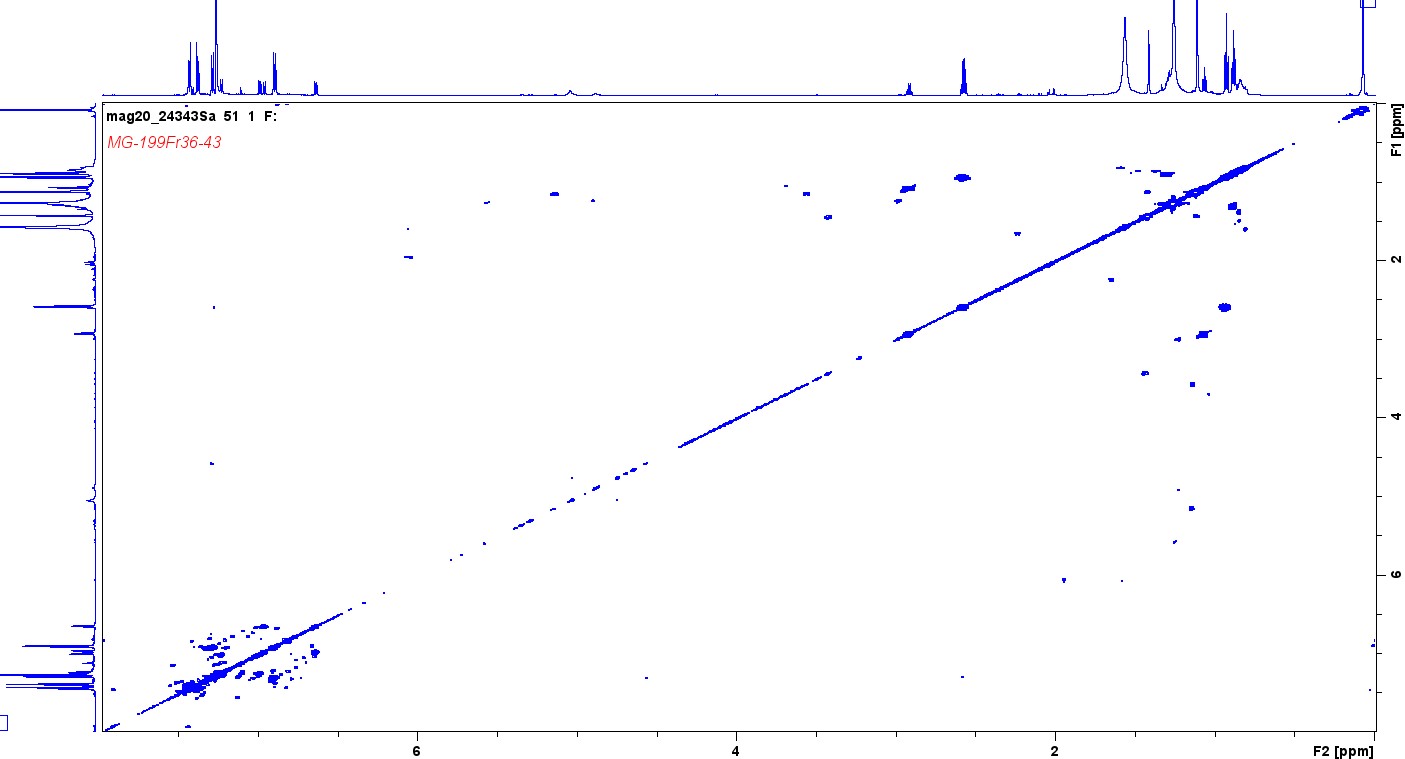


**HSQC**


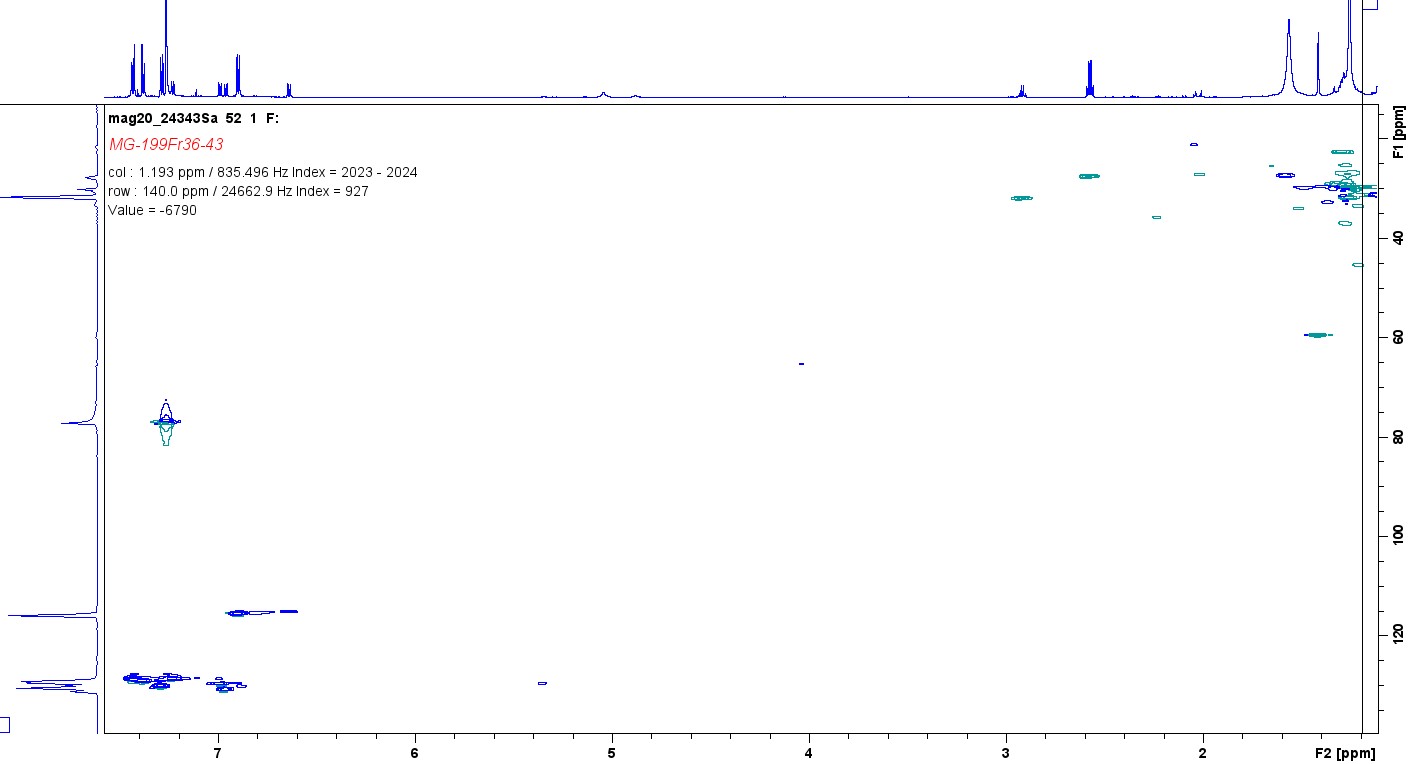


**HMBC**


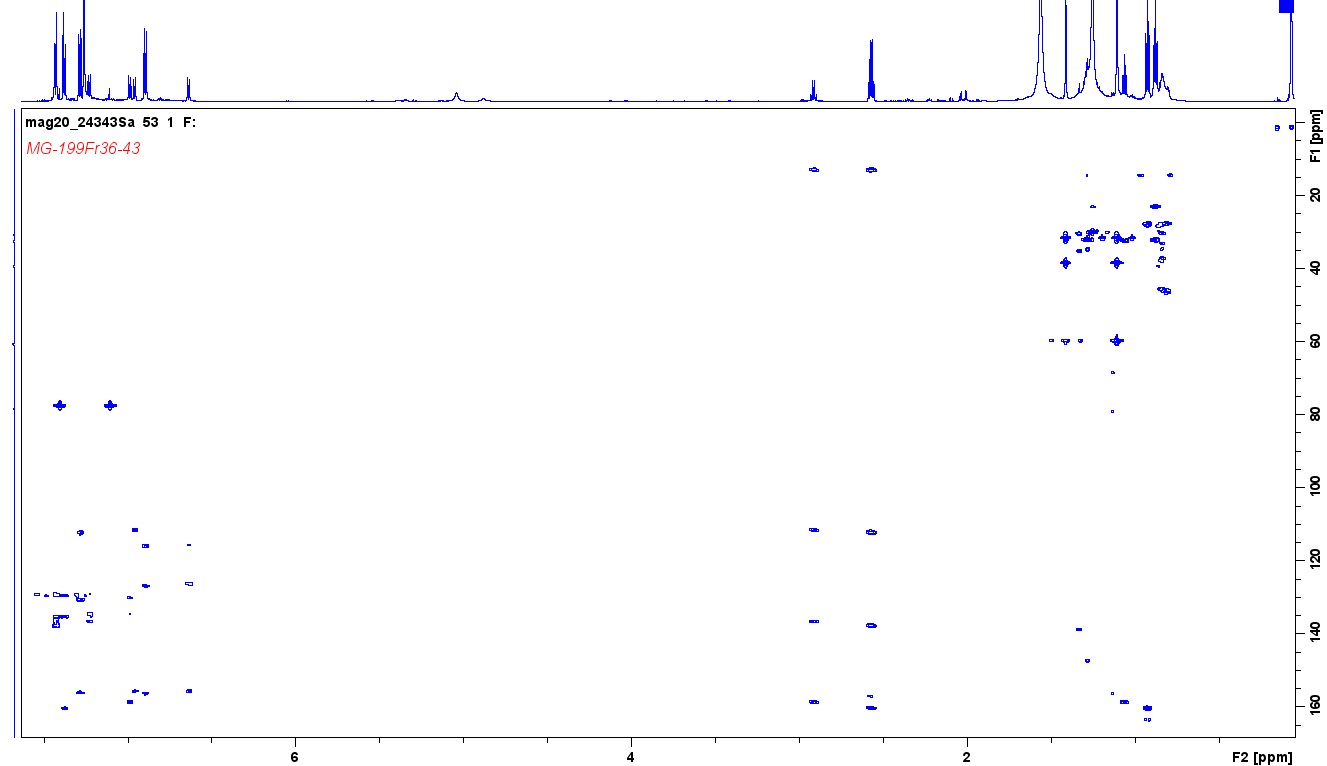


**ROESY with significant regions**

**
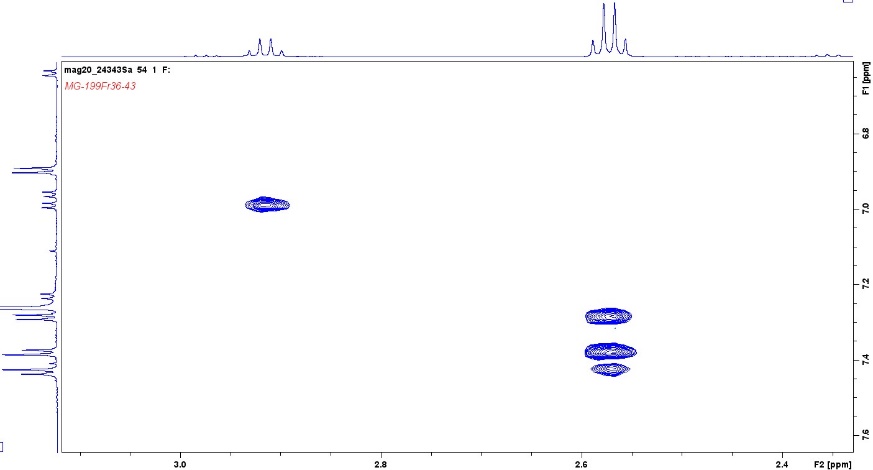
**
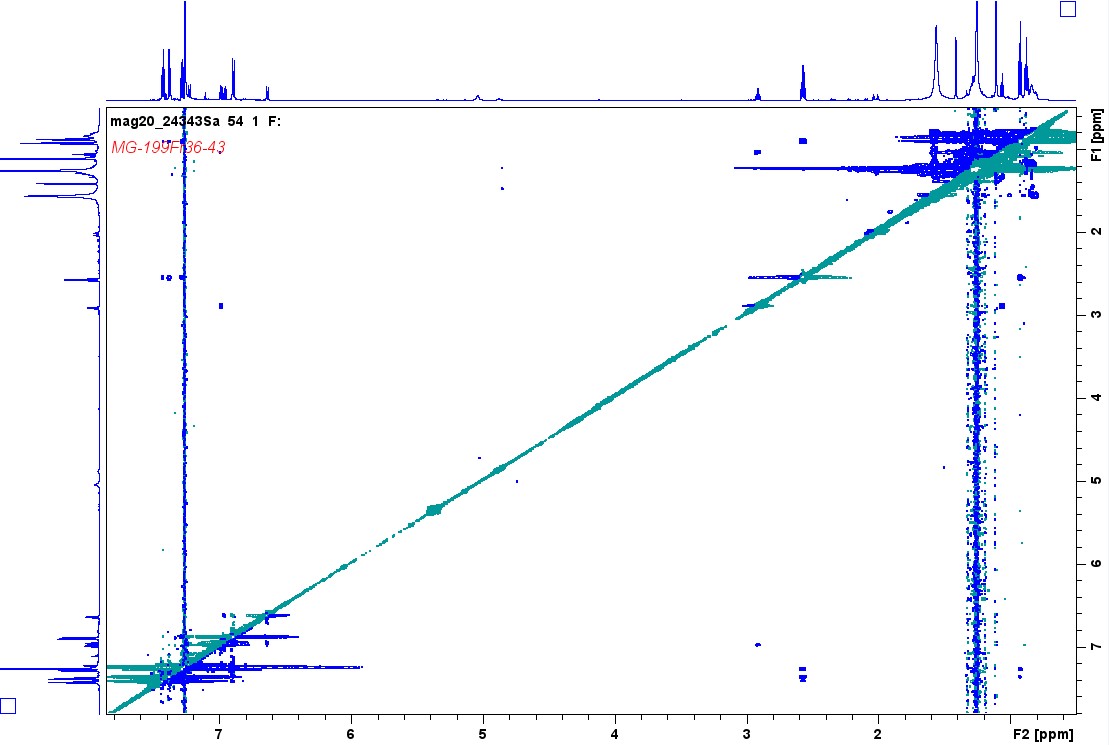


**^1^H and DEPTQ spectra of P-3622**


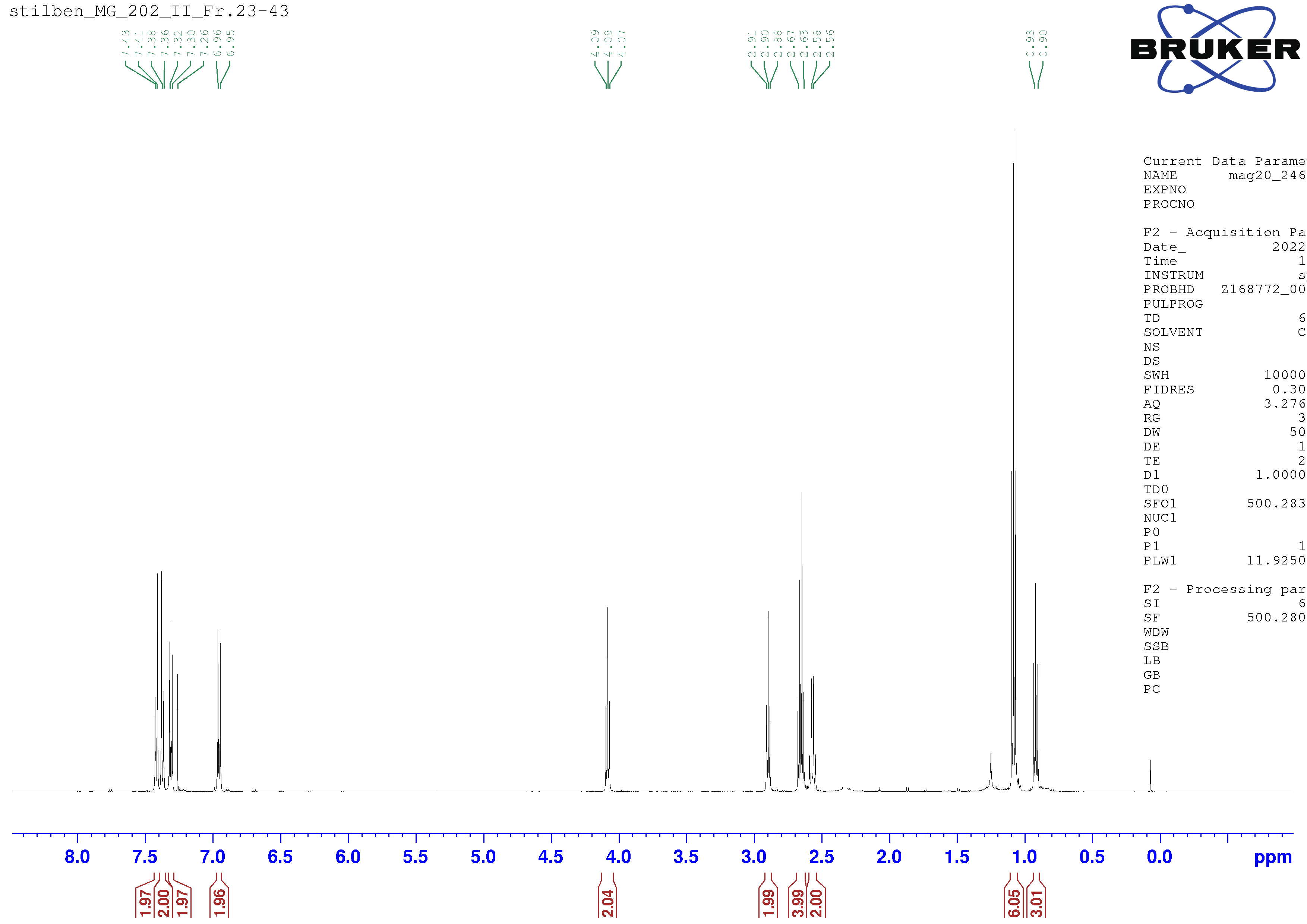


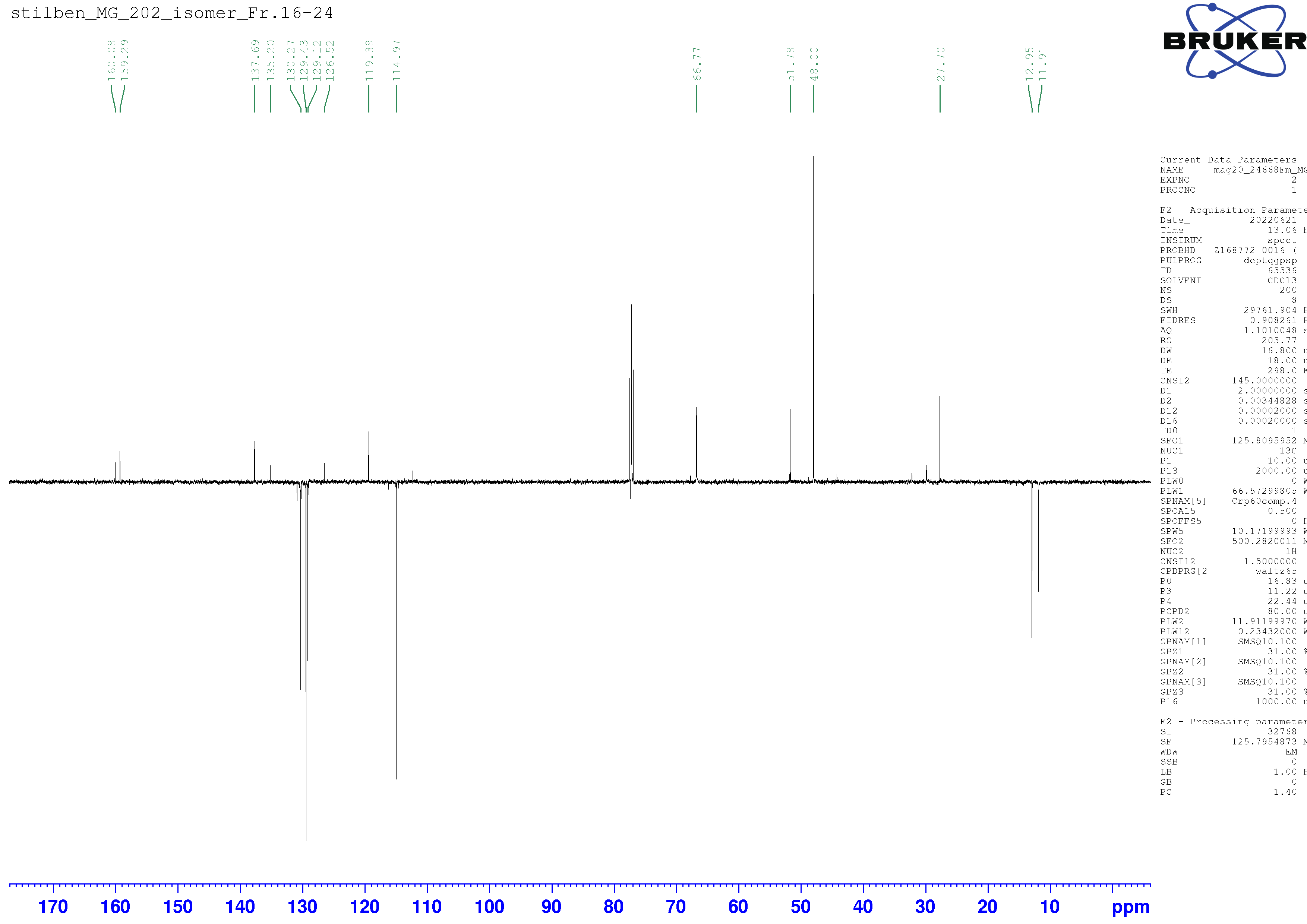


**Supplementary References**

1. Berger Rentsch M, Zimmer G. 2011. A vesicular stomatitis virus replicon-based bioassay for the rapid and sensitive determination of multi-species type I interferon. PLoS One 6:e25858.
